# A nanoencapsulated oral formulation of fenretinide promotes local and metastatic breast cancer dormancy in HER2/neu transgenic mice

**DOI:** 10.1186/s13046-024-03213-6

**Published:** 2024-11-05

**Authors:** Maria Laura De Angelis, Federica Francescangeli, Eleonora Aricò, Paola Verachi, Massimo Zucchetti, Cristina Matteo, Elena Petricci, Emanuela Pilozzi, Isabella Orienti, Alessandra Boe, Adriana Eramo, Rachele Rossi, Tiberio Corati, Daniele Macchia, Anna Maria Pacca, Ann Zeuner, Marta Baiocchi

**Affiliations:** 1 Department of Oncology and Molecular Medicine, Istituto Superiore di Sanità, Rome, Italy; 2 Department of Oncology, Istituto di Ricerche Farmacologiche Mario Negri IRCCS, Milan, Italy; 3 Department of Biotechnology, Chemistry and Pharmacy, University of Siena, Siena, Italy; 4 Department of Clinical and Molecular Medicine, Unit of Pathologic Anatomy Morphologic and Molecular, Sapienza University of Rome, Sant’Andrea Hospital, Rome, Italy; 5 Department of Pharmacy and Biotechnology, University of Bologna, Bologna, Italy; 6 Cytometry Unit, Core Facilities, Istituto Superiore di Sanità, Rome, Italy; 7 Center of Animal Research and Welfare, Istituto Superiore di Sanità, Rome, Italy

**Keywords:** Fenretinide, Breast cancer, Tumor dormancy, Cancer stem cells, Retinoids, Metastasis, Metastatic dormancy

## Abstract

**Background:**

Prevention and treatment of metastatic breast cancer (BC) is an unmet clinical need. The retinoic acid derivative fenretinide (FeR) was previously evaluated in Phase I-III clinical trials but, despite its excellent tolerability and antitumor activity in preclinical models, showed limited therapeutic efficacy due to poor bioavailability. We recently generated a new micellar formulation of FeR, Bionanofenretinide (Bio-nFeR) showing enhanced bioavailability, low toxicity, and strong antitumor efficacy on human lung cancer, colorectal cancer, and melanoma xenografts. In the present study, we tested the effect of Bio-nFeR on a preclinical model of metastatic BC.

**Methods:**

We used BC cell lines for in vitro analyses of cell viability, cell cycle and migratory capacity. For in vivo studies, we used HER2/neu transgenic mice (neuT) as a model of spontaneously metastatic BC. Mice were treated orally with Bio-nFeR and at sacrifice primary and metastatic breast tumors were analyzed by histology and immunohistochemistry. Molecular pathways activated in primary tumors were analyzed by immunoblotting. Stem cell content was assessed by flow cytometry, immunoblotting and functional assays such as colony formation ex vivo and second transplantation assay in immunocompromised mice.

**Results:**

Bio-nFeR inhibited the proliferation and migration of neuT BC cells in vitro and showed significant efficacy against BC onset in neuT mice. Importantly, Bio-nFeR showed the highest effectiveness against metastatic progression, counteracting both metastasis initiation and expansion. The main mechanism of Bio-nFeR action consists of promoting tumor dormancy through a combined induction of antiproliferative signals and inhibition of the mTOR pathway.

**Conclusion:**

The high effectiveness of Bio-nFeR in the neuT model of mammary carcinogenesis, coupled with its low toxicity, indicates this formulation as a potential candidate for the treatment of metastatic BC and for the adjuvant therapy of BC patients at high risk of developing metastasis.

**Supplementary Information:**

The online version contains supplementary material available at 10.1186/s13046-024-03213-6.

## Background

BC is the most frequent female-occurring malignancy worldwide [1]. Despite its overall outcome in patients has impressively improved over the years due to both extended methods for early diagnosis and widened therapeutic options, BC still represents the first cancer-related cause of death in women [1]. In addition to surgery and radiation therapy, BC treatment has gained efficacy from the use of targeted drugs based on tumor-specific phenotyping, including endocrine therapy and/or anti-HER2 treatment [2]. However, therapeutic options for stage IV/metastatic disease remain limited, showing a variable and temporary effectiveness in slowing tumor progression [2, 3]. Therefore, widening the array of therapeutic choices for metastatic BC represents a goal of the utmost importance. Tumor dormancy is increasingly recognized as a crucial factor influencing tumor evolution [4, 5], and understanding the biology of dormant cancer cells (DCCs) is indispensable to devise new strategies aimed at monitoring and targeting minimal residual disease [6]. In that regard, we have recently reported that lung and colorectal tumors share a common dormancy gene expression signature enriched in factors involved in stemness/self-renewal, epithelial-mesenchymal transition, TGF-β signaling, morphogenesis, cell adhesion and chemotaxis that may represent new DCCs therapeutic vulnerabilities [7]. FeR is a derivative of retinoic acid, able to induce cancer cell differentiation, growth arrest and death, through a multi-branched action on different molecular pathways linked to cell cycle, proliferation, apoptosis and stem cell dormancy [8–10]. Importantly, it shows antineoplastic activity in both preclinical and clinical settings in a variety of tumors, including pediatric neuroblastoma, melanoma, prostate, and lung cancer [11–15]. In BC, phase III clinical trials revealed significant effects of oral FeR in preventing recurrence in premenopausal women [16–18]. Notably, FeR activity was demonstrated to be independent on tumor estrogen receptors (ER) status [19, 20]. The antineoplastic effects of FeR, however, take place at relatively high blood concentrations, and the low solubility of the drug represents an important issue to solve for human treatment. In fact, in order to reach therapeutic blood concentrations, high doses and repeated administrations were required in trials, posing a serious obstacle not only to intravenous administration but also to oral delivery [11]. As an approach to this issue, we generated new FeR formulations through drug salification and complexation with solubilizing excipients, thus achieving increased bioavailability and reduced toxicity [8, 9, 21]. The first nanoencapsulated FeR formulation (nanofenretinide, nFeR) was shown to be effective against a wide panel of human malignancies both in vitro and in vivo. nFeR mechanism of action was shown to involve a combination of pro-dormancy effects (i.e. a generalized suppression of proliferative, metabolic, and biosynthetic activity) together with cytotoxicity and reduction of the stem cell compartment [8]. Subsequent drug development led to the generation of bionanofenretinide (Bio-nFeR), an improved oral formulation consisting of a nanomicellar structure in which FeR is stabilized by ion-pairing with phosphatidylcholine [9]. Our previous studies demonstrated that Bio-nFeR achieves therapeutic intratumor levels and higher drug concentration in blood as compared to FeR formulations used in clinical trials, in the absence of toxicity [9]. Bio-nFeR demonstrated mutation-independent therapeutic activity in xenografts of human melanoma, lung, and colorectal cancer, with a specific action towards cancer stem cells (CSCs) populations, i.e. ALDH1^+^ cells in lung cancer and CD44v6^+^ cells in melanoma and colorectal cancer [9]. Mechanistically, new FeR formulations were shown to inhibit cancer cell proliferation and induce ROS-dependent autophagic cell death [8, 9]. With these premises, we ought to evaluate the effect of Bio-nFeR in the neuT murine model of mammary tumorigenesis. These mice spontaneously develop mammary carcinomas that progress from focal atypical hyperplasia to in situ carcinoma, and then to invasive carcinoma, eventually generating lung metastases [22–25]. This model closely recapitulates the development of human neoplasia and, compared to the xenograft system, overcomes any potential bias deriving either from the tissue/site mismatch and/or from the impaired murine immune system [26]. Most importantly, the neuT model allows a full-spectrum analysis of metastasis development, thus providing a highly translatable model for BC drug screening. Treatment of neuT mice with Bio-nFeR resulted in a significant reduction of the number and size of primary mammary tumors with a contraction of the ALDH1^+^, CD44^+^/CD24^−^ CSCs population. Furthermore, Bio-nFeR reduced the number of lung metastases with a striking effect on metastasis size/proliferation accompanied by suppression of proliferative and biosynthetic molecular pathways. Altogether, these observations indicate a potential use of Bio-nFeR both as a chemopreventive agent and as a dormancy-inducing treatment for metastatic BC.

## Methods

### Mice

129 Sv female mice transgenic for the activated rat neuT oncogene, which spontaneously develop multiple mammary tumors, were generated as previously described [25, 27]. The BALB-neuT strain originally used for the backcrosses originated from a transgenic CD1 random-bred breeder male mouse (no. 1330) carrying the mutated rat HER-2/neu oncogene driven by the MMTV promoter [24, 25]. All mice were housed in the animal facility at Istituto Superiore di Sanità in accordance with the European Community Regulation (https://eur-lex.europa.eu/eli/reg/2019/1010/oj). At each generation, the presence of the rat HER-2 transgene was routinely checked by polymerase chain reaction (PCR) on tail DNA using primers hybridizing to vector (5-ATCGGTGATGTCGGCGATAT-3) and to MMTV sequences (5-GTAACACAGGCAGATGTAGG-3). The mammary glands of all transgenic virgin female mice were inspected once a week for tumor appearance. Progressively growing masses with a mean diameter > 1 mm were regarded as tumors. Individual neoplastic masses were measured with a caliper in two perpendicular diameters and tumor volume was calculated according to the formula: V = 1/6 π x D x d x [(D + d)/2] [28], where d and D represent shorter and longer tumor measurements, respectively. Tumor multiplicity was calculated as the cumulative number of incident individual tumors/total number of mice and reported as mean ± standard error. Bio-nFeR freshly dissolved in sterile water was administered by oral gavage 100 mg/kg, 5 days/week, from week 14 to week 32 post-birth (PB), or to euthanasia. Mice were examined twice a week for signs of distress and were euthanized when 10/10 mammary glands developed tumors. All surviving mice were euthanized at week 32 PB for ethical reasons related to repeated gavage procedures. At the end of the experiments, mice were sacrificed by cervical dislocation, and tumors harvested ex vivo were measured and weighed. Fragments were collected from each tumor for subsequent analyses or storage. All the procedures were approved by the Ethics Committee for Animal Experimentation of the Istituto Superiore di Sanità, according to the Italian regulation (DL 4.3.2014 N. 26). For the second transplantation assay, cells were dissociated from individual tumors and pooled for each mouse. Cell pools generated from single mice were then transplanted subcutaneously into secondary recipient NOD.Cg-Prkdc scid Il2rg tm1Wjl /SzJ (NSG) mice. Each treatment group included cell pools generated from six individual mice. For each cell pool, different cell doses (1, 10, 100 and 1000) were tested. Mice were recorded negative when no graft was observed after 24 weeks from the inoculation. CSC frequency was calculated by the extreme limiting dilution analysis software ELDA [29].

### Plasma fenretinide determination

The quantification of fenretinide (4-HPR) and its main metabolites, O-methylated (4-MPR), 4-oxo-substituted β-ionone ring (4-oxo-4HPR ), dehydrogenated 4-HPR (DH-4HPR ), was performed according to [30].

Shortly, 30 µL aliquot of plasma was added with 3 ng of deuterated internal standard (2H4 4-HPR), deproteinized by 90 µL of acetonitrile and centrifuged 5 min at 13,200 rpm at 4 °C. The supernatant was recovered and 8 µL injected into a HPLC system (1200 series pump and auto sampler Agilent Technologies, Santa Clara, CA, USA). Chromatographic separation was achieved on a Gemini-C18 column (50 mm × 2.0 mm, 5 μm particle size; Phenomenex Inc., Torrance, CA, USA) at 35 °C, protected with a Security Guard™ ULTRA cartridges C18. The detection, obtained via high-resolution mass spectrometry (HRMS), was carried out on high-resolution LTQ-Orbitrap XL mass spectrometer (Thermo Scientific Inc., Waltham, MA, USA), equipped with an electrospray source (ESI) operating in positive ion mode.

### Cell lines

MCF7, generated from a human invasive breast ductal carcinoma, ER-positive [31] and MDA-MB-231, generated from human breast adenocarcinoma, ER- and PR-negative [32] cell lines were purchased from ATCC (Manassas, VA, USA). TUBO, a murine mammary tumor cell line cloned from a BALB-neuT mouse mammary carcinoma [33] was from Sigma Aldrich (St. Louis, MO, USA). Cells were cultured according to the manufacturers’ instruction, and used within the 10th passage.

### Antibodies and reagents

Fenretinide (code 65646-68-6) was purchased from Olon (Milan, Italy). L-α-phosphatidylcholine from egg yolk, glyceryl tributyrate, and all the other chemicals were purchased from Sigma-Aldrich. Anti-β-tubulin and anti-β-actin antibodies were purchased from Sigma-Aldrich; Phospho-S6 Ribosomal Protein (Ser240/244) #2215 and S6 Ribosomal Protein (5G10) Rabbit Ab #2217, Caspase-3 Rabbit Ab #9662, Caspase-7 (D2Q3L) Rabbit Ab #12,827, Phospho-p38 MAPK (Thr180/Tyr182) (D3F9) XP^®^ Rabbit Ab #4511, p38 MAPK (D13E1) XP^®^ Rabbit Ab #8690, mTOR Rabbit Ab #2972, Phospho-mTOR (Ser2448) Rabbit Ab #2971, 4E-BP1 (53H11) Rabbit Ab #9644, Phospho-4E-BP1 (Ser65) Rabbit Ab #9451, Bcl-2 (D17C4) Rabbit Ab #3498, ALDH1 (D4R9V) Rabbit Ab #12,035, and Ki67 (D3B5) Rabbit Ab (IHC Formulated) #12,202, Ki67 (D3B5) Rabbit Ab #9129, PCNA (D3H8P) Rabbit Ab #13,110 were from Cell Signaling Technology (Danvers, MA, USA). Anti-CDKN2A (p16, 15C10C30) was from Biolegend (London, United Kingdom). Cyclin D1 (A-12) Mouse mAb #sc-8396, ERK-1 (K-23) Rabbit pAb #sc-94, p-ERK (E-4) Mouse mAb #sc-7383 were from Santa Cruz Biotechnology (Texas, USA). Secondary anti-mouse and anti-rabbit antibodies coupled to horseradish peroxidase were from Bio-Rad (Hercules, CA, USA). Alexa Fluor 647 conjugated anti-Ki67 (cloneB56), FITC conjugated anti CD24 (clone M1/69), PE conjugated anti-CD44 (clone IM7) were from BD Pharmingen (San Diego, CA).

### Bio-nFeR preparation

Bio-nFeR lot preparation and quality control are described in detail in [9]. Briefly, FeR was homogeneously mixed with L-α-phosphatidylcholine and glyceryl tributyrate dispersed in alkaline ethanol to a final weight ratio of 1:9:1 w: w:w, respectively. Ethanol was then removed from the mixture by a rotary evaporator and the dry residue was stored at -20 °C until use. Reconstitution of the dry residue to Bio-nFeR nanomicelles was accomplished by dissolving the residue in water at 30 °C in an ultrasound bath with a wave frequency of 40 kHz. The dissolved phase (100 mg/mL) was subsequently filtered through 0.4 μm pore filters to obtain homogeneously dispersed nanomicelles of controlled mean size. Characterization of the Bio-nFeR preparation was performed by multiple assays including fluorescence microscopy (to detect auto-fluorescent nanomicelles containing FeR), dynamic light scattering, drug loading, solubilization, drug release from the nanomicelles over time [9]. Bio-nFeR lot was divided in batches, which were aliquoted and stored at -20 °C. Before use, each Bio-nFeR frozen batch was thawed and diluted with sterile water to the desired concentrations (see Results). As for our routine procedure, the batch was tested after thawing by assessing its biological activity on two freshly thawed lung spheroid lines, in comparison with published reference data [9] (Additional file: Fig. S1).

### Viability assay

Cell viability was determined by CellTiter-Glo luminescent cell viability assay (Promega, Madison, WI, USA) according to the manufacturer’s directions. Briefly, cells were detached from flasks by trypsin incubation at 37 °C (Thermo Fisher Scientific) and seeded in 96-well plates (3 × 10^3^/well, three replicates per experimental point), in culture medium. Dishes were incubated in a humidified atmosphere at 37 °C, 5% CO_2_. Cells were treated with Bio-nFeR at different concentrations (from 5µM to 100µM) as indicated in the Results, and then analyzed after 72 h. Luminescence was detected by a DTX880 multimode microplate reader (Beckman Coulter, Brea, CA, USA).

### Flow cytometry and cell cycle

For flow cytometry, cells (either cell lines detached from flasks by short treatment TrypLE Express (Thermo Fisher Scientific), or cells dissociated from primary tumors by mechanical/enzymatic treatment with TrypLE Express), were resuspended in PBS (5 × 10^5^/ml), 0.4% BSA/0.5 M EDTA, and labeled with antibodies (PE-anti CD44, FITC-anti CD24 and Alexa Fluor 647-anti Ki67, see Antibodies and Reagents section in Methods), for 1 h on ice.

For CD44/CD24 detection, the analysis was preceded by depletion of mouse hematopoietic cells with the Mouse Lineage Cell Depletion Kit (Miltenyi Biotec, Bergisch Gladbach, Germany), to obtain lineage-negative (LIN^neg^) cell populations. Marker analyses (CD44/CD24 and Ki67) were performed by a FACSCanto flow cytometer equipped with a DIVA software (Beckton Dickinson). Cell population was gated based on FS (forward scatter) and SC (side scatter) properties, to exclude debris. SSC-A vs. SSC-W were then plotted to exclude doublets, and dead cells were excluded by staining with the viability dye 7AAD (7-amino-actinomycin D, Sigma-Aldrich). The cells within the viability gate were further analysed for marker(s) expression. At least 20.000 single cells were collected per sample.

The cell cycle status of BC cell lines was assessed by staining freshly detached single cells with 50 mg/ml propidium iodide (PI) dissolved in 0.1% trisodium citrate buffer, 9.65 mM NaCl, 0.1% NP40, 200 mg/ml RNAse for 1 h at room temperature. The analysis was performed by a Cytoflex LX flow cytometer (Beckman Coulter), using yellow laser (561 nm). Acquisition was done at a low flow rate under 300 events/second. At least 20.000 single cells/sample were recorded. Cell population was gated by FS (forward scatter) and SC (side scatter) analysis. PI emission was then measured by the bandpass 610/20 nm filter. The gated population was plotted for PI area versus PI width to identify cell doublets and clumps, which were gated out. PI was plotted on a linear scale to clearly distinguish the cell cycle phases. The PI histogram graph of this gated population shows the three distinct phases of the proliferating cell population: G0/G1, S and G2 /M. The percentage of cells in each cell cycle phase was quantified by using markers set within the analysis, which was conducted using the CyExpert software (Beckman Coulter).

### Western blotting

Fragments of frozen tissues (~ 50 µg) were lysed in lysis buffer (10 mM Tris pH8, 150 mM NaCl, 60 mM Octyl-β-Glucoside, supplemented with protease inhibitor/phosphatase inhibitor cocktails I and II from Sigma-Aldrich). Tissue homogenization was performed with Pro 200 Kema Keur (Pro Scientific Inc. Oxford) at max speed, at 4°C, for 30”. Lysate’s concentration was determined by Bradford assay (Bio-Rad Laboratories, Hercules). Equal amounts of proteins were run on a 4–12% precast gel (Thermo Fisher Scientific) and then transferred to nitrocellulose membranes (GE Healthcare Life sciences). Blots were blocked with TBST 5% non-fat dry milk (Bio-Rad Laboratories, Hercules, CA, USA) and incubated overnight at 4 °C with primary antibodies (described in the Antibodies and Reagents section) diluted in TBST/BSA 5%. After three washes in TBST, blots were incubated for 45 min with specific secondary HRP-conjugated antibodies dissolved in TBST, 5% BSA. Chemiluminescent signals were detected with Amersham ECL Prime or Select western blotting detection reagent (GE Healthcare Life Sciences, Barrington, IL, USA). Immunoblotting images were recorded and analyzed by Bio-Rad ChemiDoc Imagers (Bio-Rad Laboratories, Hercules). Immunoblot densitometry quantification was performed by ChemiDocMP (Bio-Rad Laboratories, Hercules) and signal intensity was quantified with the Image Lab software. Normalization was performed using antibodies against β-actin or β-tubulin (both from Sigma-Aldrich) as reference standards. The antibodies used for Western blotting are indicated in the antibodies and reagents section.

### Migration/Invasion assay

Single cells (MCF7, TUBO, and MDA-MB-231, 1.5 × 10^4^/experimental point) were suspended in 200 µl of serum-free medium and plated in Matrigel^®^ into the upper wells of Boyden Chambers containing porous 8 μm diameter polycarbonate membranes (Costar Scientific Corporation). Lower wells contained 500 µl of complete medium (10% FBS). Bio-nFeR (20µM, 40µM and 80 µM) was added into the upper wells. After 48 h, upper wells were removed, and cells migrated to the lower wells were fixed in 4% paraformaldehyde (PFA), stained with DAPI in PBS 1% NP40 for 5 min, and counted under a fluorescence Zeiss Axio Scope.A1 microscope equipped with a 10x objective. The number of migrated cells was quantified by the ZEN 2.6 software (blue edition).

### Scratch test

MDA-MB-231, TUBO and MCF7 cells were seeded into 6-well tissue culture plates, and incubated at a density optimized to reach confluency after growing overnight at 37 °C. The following day, confluent cell monolayers were scraped in a straight line with a p200 pipet tip to create the scratch. Debris were removed by washing the cells with PBS and then replicate wells were filled with 4 mL of fresh medium, in the presence or the absence of 20 µM Bio-nFeR. Cell migration was monitored by time-lapse imaging using a Zeiss LSM900 confocal microscope. For MDA-MB-231 cell line, images were taken at a 10 min time intervals up to 24 h. For MCF7 and TUBO cells, images were taken at a 20 min time intervals up to 48 h. Three images for each time point were then analyzed with ImageJ by using a specific plugin (https://github.com/AlejandraArnedo/Wound-healing-size-tool/wiki), which calculates the average distance (width) between the edges of the scratch on each image. Data represent the mean percentage of width change over time ± SD, on the three images taken for each time frame.

### Colony formation assay

Clonogenic units present in xenograft-dissociated cells were assessed by plating 500 cells/ml per well in triplicate in 24-well plates containing a soft agar bilayer (0.3% top and 0.4% bottom layer; SeaPlaque Agarose; Cambrex). Cultures were incubated in humidified atmosphere at 37 °C and 5% CO_2_ for 21 days. Colonies were stained with crystal violet (0.01% in 10:1 methanol to water), and counted under a light microscope. Data represent the percentage of colonies normalized to the number of single cells counted the day after plating.

### Histology

Tumors and lungs ex vivo were fixed for 18–24 h in 10% buffered formalin immediately after removal. After fixation, samples were processed, and embedded in paraffin, sectioned and stained by H&E. To evaluate metastases, each lung was put in a separate tissue cassette, and then 3 sections, 2 μm thick at 2 mm intervals, were cut from every sample. H&E-stained slides were then scanned with Aperio C2 Pathology Scan (Leica) to obtain high quality digital slides. Metastases were then counted and measured. Digital pictures (1x) were obtained by using Aperio ImageScope program.

### Immunohistochemistry

For immunohistochemistry, 5 μm sections were deparaffinized in xylene and rehydrated in a series of graded ethanol washes. Endogenous peroxidase activity was blocked with 3% H_2_O_2_ for 10 min. Antigen retrieval was performed by incubation with citrate buffer (pH 6.0) for 20 min at 100 °C followed by cooling at room temperature for 30 min. Slides were then incubated with anti-Ki67 (#9129, Cell Signaling Technology) and anti-PCNA (Proliferating Cell Nuclear Antigen) (#13110, Cell Signaling Technology) antibodies overnight at + 4 °C and for 1 h at 37 °C, respectively. Immunoreactions were detected with EnVision Detection SystemsPeroxidase/DAB, Rabbit/Mouse kit (#K5007, DAKO, Agilent Technologies), following manufacturer’s instructions. Slides were counterstained with Mayer’s hematoxylin (#MHS32, Sigma Aldrich), dehydrated, and mounted with Canada balsam mounting medium (#C1795, Sigma Aldrich). Images were acquired with a Zeiss Axio Scope.A1 Microscope equipped with 5x and 20x objectives. Image analysis was performed using the software ZEN 2.6 (blue edition). The proliferation index was calculated within the region of interest (ROI) by computer-assisted imaging with the ZEN 2.6 software (blue edition), as the ratio between the pixel count for Ki67- or PCNA-positive nuclei, over the pixel count for total nuclei staining (hematoxylin-positive).

### Immunofluorescence and confocal analysis

Paraffin-embedded section of primary tumor tissue samples were deparaffinized, rehydrated and treated with citrate buffer as described in the previous paragraph. Slides were then quenched with 1 M glycine in PBS and incubated overnight at 4 °C with primary antibody anti-Ki67 (#9129, Cell Signaling Technology). After washing in PBS, sections were incubated with secondary antibodies (see Antibodies and Reagents section) for 45 min at room temperature in the dark. Terminal deoxynucleotidyl transferase dUTP nick end labeling (TUNEL) assay was performed using in situ cell death detection KIT fluorescein (Roche-12156792910) following the manufacturer’s instructions. Nuclei were counterstained with DAPI (Invitrogen) for 15 min at room temperature. Slides were permanently mounted with Prolong-Gold Antifade (Thermo Fisher) and analyzed using a Zeiss LSM900 Confocal microscope equipped with a 60x oil immersion objective.

Human MCF7, MDA-MB-231 and murine TUBO cells were seeded into multi-wells on poly-L-lysine-coated coverslips and treated for 24 h with Bio-nFenR at different concentrations for each line, as indicated in the Results. After washing with PBS, cells were fixed with PFA 4% for immunofluorescence analysis. Staining with anti-Ki67 antibody was performed as described above. Nuclei were counterstained with DAPI (Invitrogen) for 15 min at room temperature. Slides were permanently mounted with Prolong-Gold Antifade (Thermo Fisher) and analyzed using a Zeiss Axio Scope.A1 Microscope equipped with 20x objective.

The proliferation index was calculated within the region of interest (ROI) by computer-assisted imaging with the ZEN 2.6 software (blue edition), as the ratio between the pixel count for Ki67-positive nuclei, over the pixel count for total nuclei staining (DAPI-positive).

### Statistical analysis

Statistical analyses were performed using GraphPad Prism version 4.0 for Windows (GraphPad Software). Statistical significance is expressed as *, *p* < 0.05, **, *p* < 0.01 and ***, *p* < 0.001. Results are presented as the mean ± SD or mean ± SEM where appropriate. Unpaired Student’s t-test was used for group comparison. IC50 was calculated according to the formula Y = Bottom + (Top-Bottom)/(1 + 10LogEC50X) by GraphPad. The survival test was analyzed by Long-rank (Mantel-Cox) test.

## Results

### Bionanofenretinide inhibits BC cell proliferation and migratory capacity

**Fig. 1:**
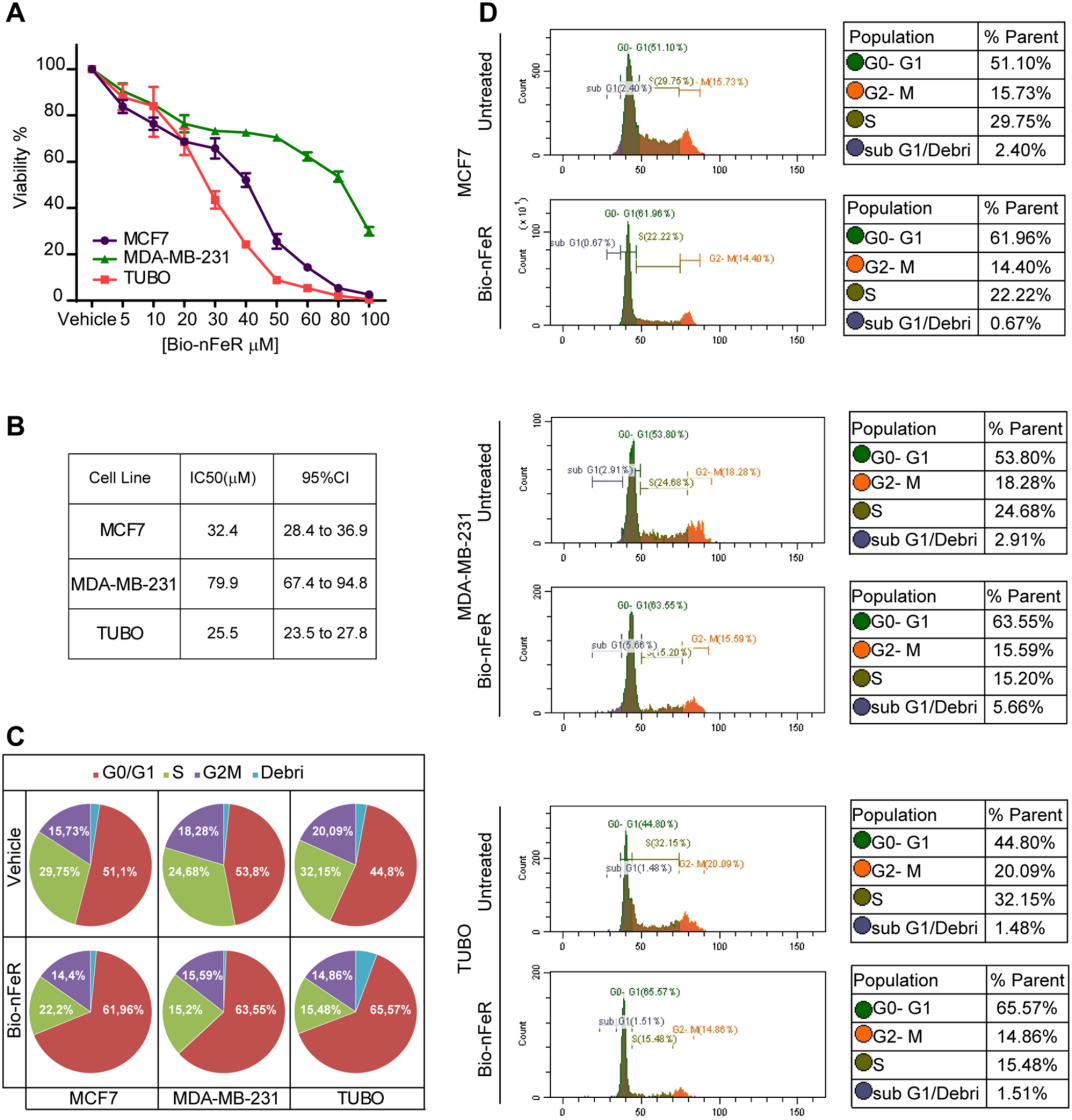
In vitro test of Bio-nFeR on human and murine BC cells. **(A)** Cell viability of BC commercial cell lines, human MCF-7 (purple) human MDA-MB-231 (green), and murine TUBO (red) treated with Bio-nFeR at the indicated concentrations for 72 h. Values represent the mean ± SD of three independent experiments. **(B)** IC50 of Bio-nFeR determined in BC cell lines indicated in A. **(C)** Cell cycle determination of Bio-nFeR-treated cell lines, as described in the Methods section. **(D)** Representative cell cycle analysis plots

**Fig. 2:**
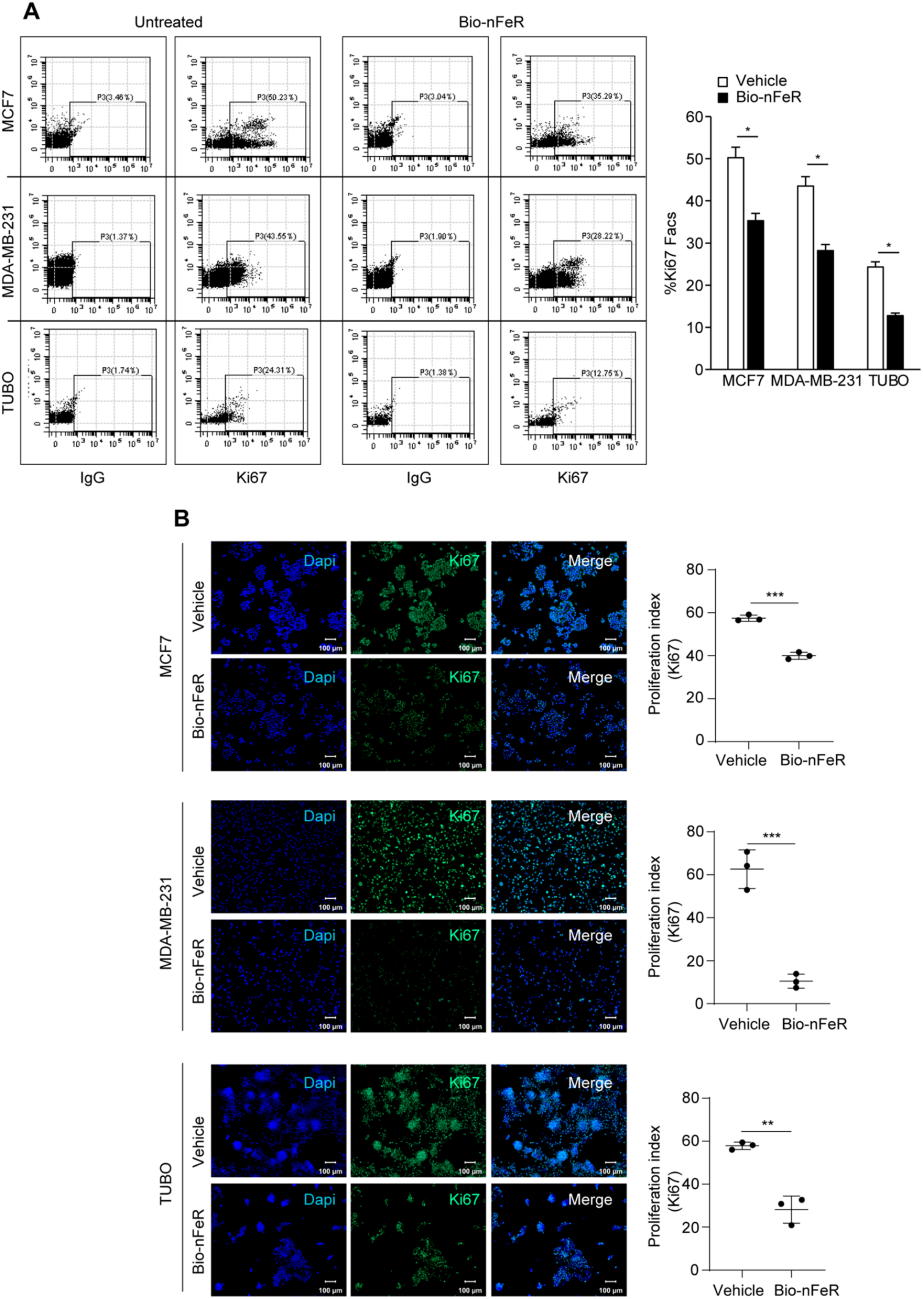
Proliferative activity of Bio-nFeR-treated human and murine BC cells. **(A)** Flow cytometry analysis for the frequency of Ki67-positive cells in MCF7, MDA-MB-231 and Tubo lines. Cells were treated with 30µM, 80µM, 25µM Bio-nFeR, respectively, for 24 h. **(B)** Left: Representative confocal images of Ki67-positive nuclei of BC cell lines treated as in A; Right: Proliferation index was calculated as the ratio Ki67-positive/ total nuclei by automated pixel counting, see Methods. Scale bar 100 μm

**Fig. 3:**
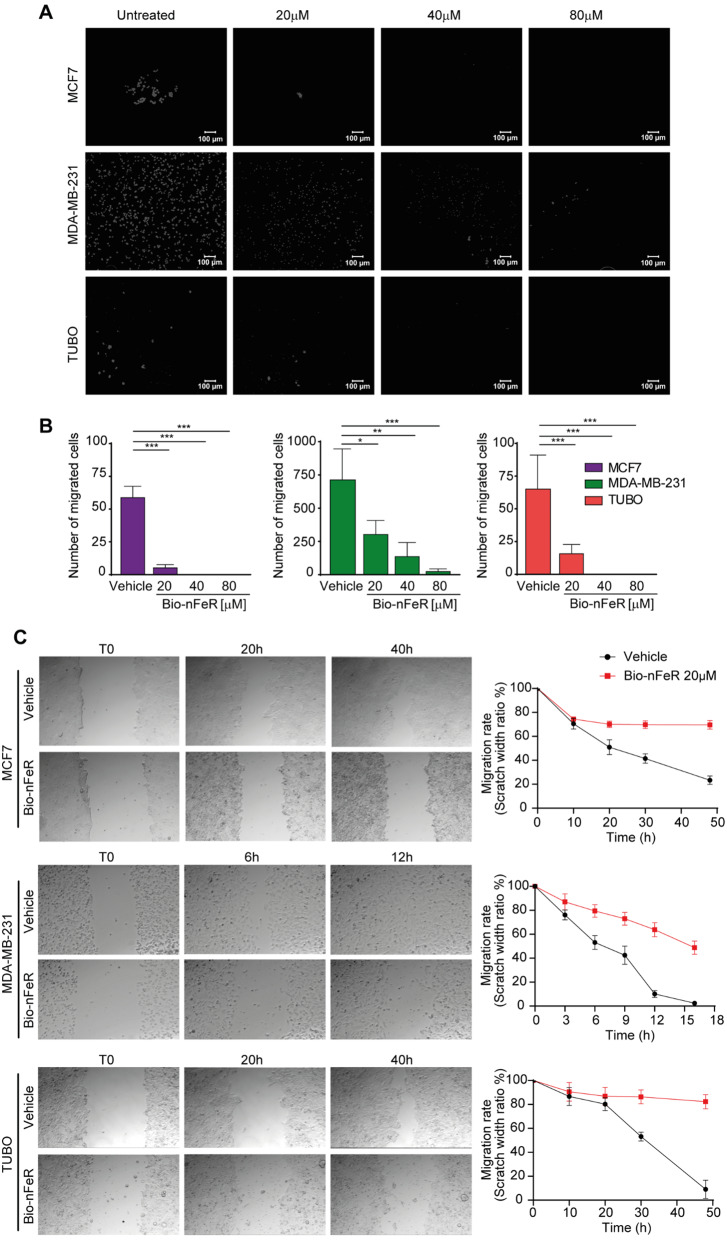
Migration and invasion activity of Bio-nFeR-treated human and murine BC cells. **(A)** Representative images of Matrigel invasion assay on cells treated with Bio-nFeR 20–80 µM for 48 h. **(B)** Graphs indicating the number of migrated cells. Values represent the mean ± SD of three independent experiments. *P < 0.05, **P < 0.01, ***P < 0.001 by unpaired Student’s t test. **(C)** Left: Scratch test of cells in the presence of Bio-nFeR 20 µM up to 48 h, as detailed in Methods; Right: Graphs indicating the relative scratch width reduction over time. Values represent the mean ± SD of three technical replicates

Bio-nFeR was previously shown to be therapeutically effective against lung cancer, colorectal cancer, and melanoma both in stem cell-enriched primary cultures and in tumor-derived xenografts [9]. To assess the effect of Bio-nFeR on BC cells in vitro, we treated three BC cell lines with increasing concentrations of the drug. Specifically, we used human MCF7 cells, expressing the estrogen receptor [31], human MDA-MB-231, characterized by a triple-negative aggressive phenotype [32], and murine TUBO cells, generated from a BALB-neuT adenocarcinoma [33]. As shown in Fig. 1A, Bio-nFeR exerted a dose-dependent effect on both human and mouse cells, resulting in a progressive decrease of ATP consumption. Importantly, lower (5µM) drug doses effectively decreased the amount of metabolically active cells, in line with previous results showing that corresponding intratumor levels of Bio-nFeR exerted a therapeutic effect in vivo [9]. Higher doses of Bio-nFeR induced cytotoxicity in the three cell lines - although with different IC50 - in agreement with previous reports showing an ER-independent effect of FeR on BC status [19, 20] (Fig. 1B and Additional file; Fig. S2). Notably, Bio-nFeR was effective also against MDA-MB-231 (with a higher IC50 as compared to the other two cell lines tested) despite the highly aggressive phenotype of these cells. Then, we analyzed the effect of Bio-nFeR on the cell cycle (Fig. 1C and D). Treatment significantly increased the frequency of cells in the G0/G1 phase, with a consequent reduction of the G2/M and the S fractions, in all the cell lines tested. By contrast, the proportion of dead cells/debris did not increase consistently, indicating that the prevalent effect of Bio-nFeR is cell cycle arrest/slowdown rather than cell death. Consistently, flow cytometry, and immunofluorescence analysis of Ki67 showed a lower frequency of proliferating cells in treated versus untreated tumor cells (Fig. 2A-B). To evaluate the effect of Bio-nFeR on the motility and migratory ability of BC cells, we first performed a Matrigel^®^ invasion assay. As shown in Fig. 3A and B, Bio-nFeR inhibited the migration of TUBO (red, right panel), MCF7 (purple, left panel), and MDA-MB-231 cells (green, middle panel), with a lower (but still significant) effect in the latter cell line. To further verify this result, we then performed a scratch test on the three cell lines, which confirmed a reduction in cell migration and invasion capability in the presence of Bio-nFeR (Fig. 3C and Additional videos 1–6). Altogether, in vitro analyses of the Bio-nFeR effect on BC cell proliferation and migration supported further evaluations of the drug’s biological activity on breast tumors in vivo.

### Bio-nFeR inhibits the initiation and progression of BC in neuT mice

**Fig. 4:**
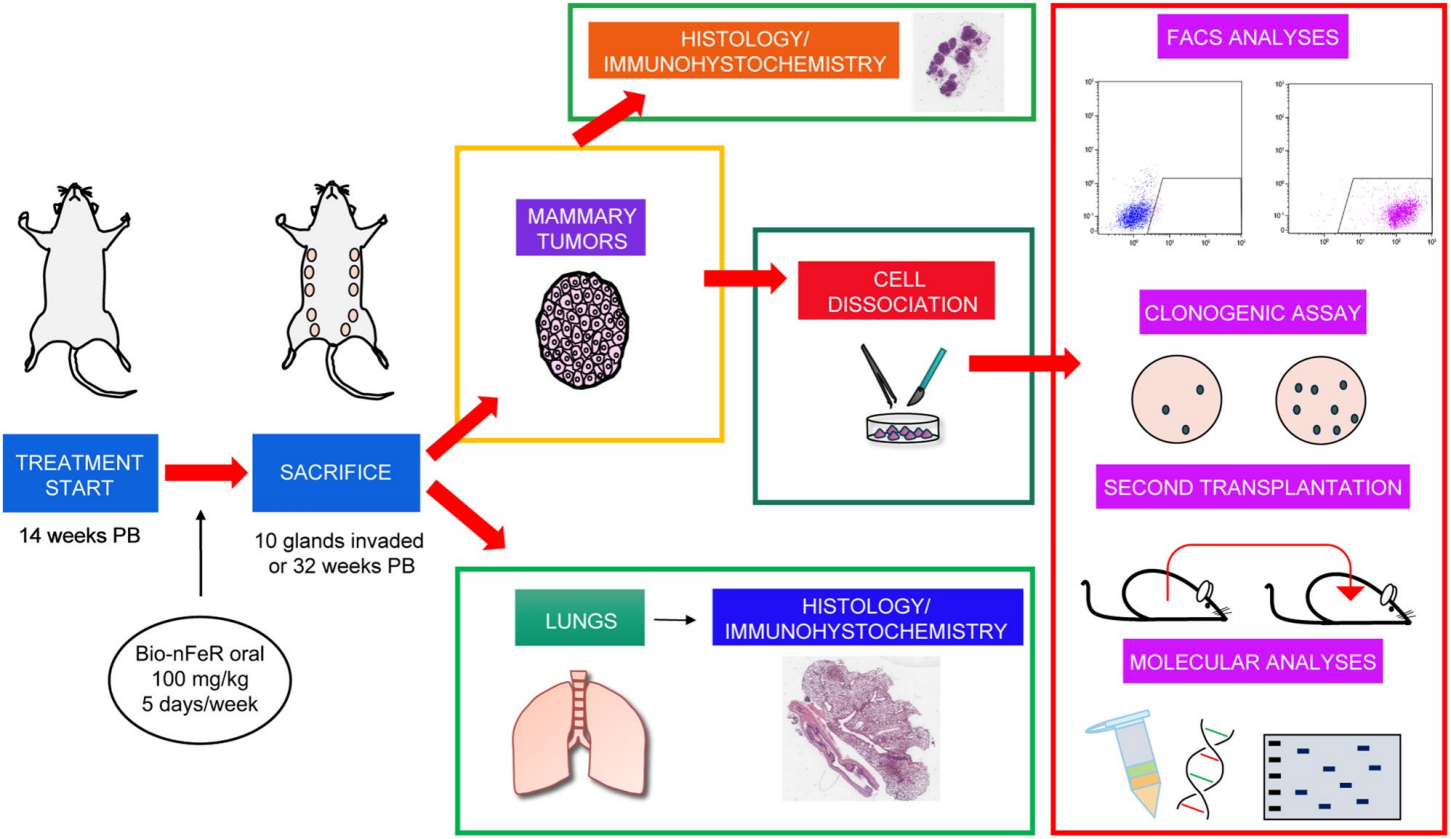
Workflow of in vivo experiments. NeuT mice were administered with Bio-nFeR from week 14 to week 32 post-birth (PB) as described in the Methods section. At sacrifice, tumors and lungs were harvested. From each mouse, two tumors were utilized for immunohistochemistry. The remaining tumors were divided into fragments. Tissue fragments were snap frozen to be later used for molecular analyses, or dissociated into single cells to perform flow cytometry analyses, agarose clonogenic assay, or second transplantation into NSG mice. Lungs were processed for histological analysis to detect metastases’ presence, frequency, and size

**Fig. 5:**
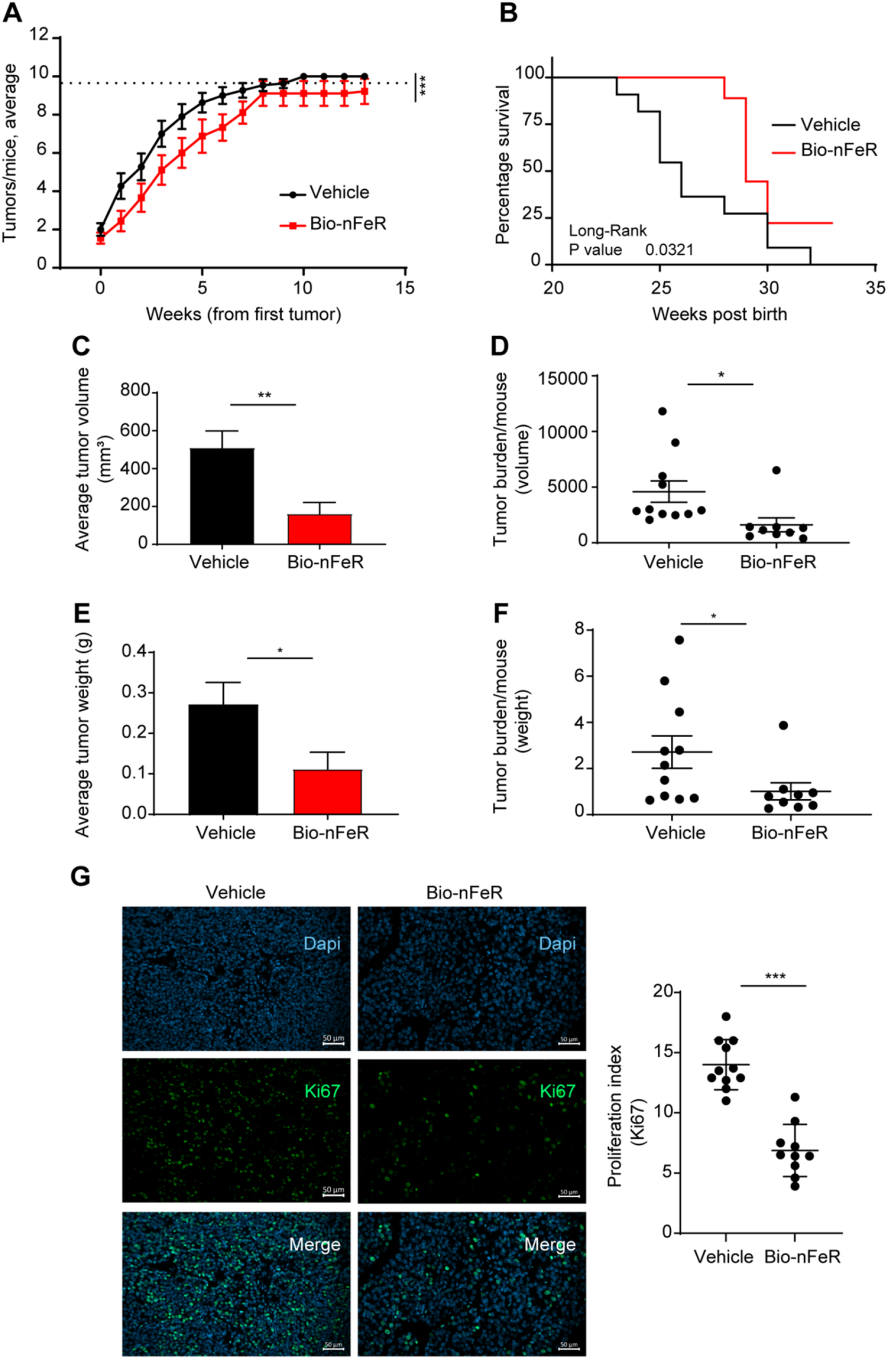
In vivo test of Bio-nFer on neuT mice. Bio-nFeR was administered at 100 mg/kg daily, 5 days/week, from week 14 to week 32 PB by oral gavage. **(A)** Time-course of tumor development in the mammary glands over time, post first tumor occurrence in vehicle (black) versus Bio-nFeR (red) mice. **(B)** Time to reach 10/10 mammary gland invasion in vehicle (black) versus Bio-nFeR (red) mice, post birth. **(C)** Average tumor volume at sacrifice in vehicle (black) versus Bio-nFeR (red) mice. **(D)** Scatter plot of average tumor burden (volume) at sacrifice in Bio-nFeR versus control mice. **(E)** Average tumor weight at sacrifice in vehicle (black) versus Bio-nFeR (red) mice. **(F)** Scatter plot of average tumor burden (weight) at sacrifice in vehicle versus Bio-nFeR mice. **(G)** Left: representative images of immunofluorescence staining of mammary tumors harvested ex vivo from vehicle-treated and Bio-nFeR-treated mice (20x, 0,7x zoom magnification, scale bar 50 μm). Ki67 (pseudocolored in green) and DAPI nuclear staining (blue); Right: proliferation index by Ki67 nuclear staining on sections. Data in **A** represent Mean ± SEM, ***P < 0.001 by paired Student’s t test with Wilcoxon test. Data in B were analyzed by Long-rank (Mantel-Cox) test, *P < 0.05 and. Data in **C**,** D**,** E**,** F** represent Mean ± SEM, *P < 0.05 and **P < 0.01 by unpaired Student’s t test with Welch correction. Data in **G** represent Mean ± SD, *P < 0.05 by unpaired Student’s t test with Welch’s correction

To assess the effect of Bio-nFeR on BC in vivo, we employed neuT mice, which express a constitutively activated form of the rat HER2/neu oncogene under the MMTV promoter ensuring the spontaneous development and metastatization of mammary tumors. The workflow of in vivo experiments with Bio-nFeR is summarized in Fig. 4. Mice were administered 100 mg/kg Bio-nFeR by oral gavage daily, 5 days/week starting at week 14 post-birth (PB) when palpable tumors are not yet detectable. Treatment was extended until tumors developed in all the ten mammary glands, which represents the euthanasia point. Surviving mice were sacrificed at week 32 PB for ethical reasons to reduce the risk of discomfort due to repeated gavage procedures and to age-related cumulative effects of tumor burden. Mice were monitored for apparent signs of toxicity (measured as weight loss, fur loss, and hunched posture) twice a week during all the experiments. No signs of suffering unrelated to tumor growth were observed. In addition, no liver or blood toxicity was evident by the analysis of liver enzymes and hematological parameters after acute treatment for 2 weeks with 100 mg/kg Bio-nFeR (Additional file: Tab. S1A and B). Analysis of fenretinide plasma concentrations in the same mice in turn showed results comparable to those achieved by Bio-nFeR in our previous studies (Additional file: Tab. S1C). These results altogether confirm our previous observations that nanoencapsulated FeR formulations achieve a high bioavailability in the absence of relevant toxic side effects [9]. Bio-nFeR-treated mice displayed a delay in tumor occurrence (Fig. 5A); indeed, at sacrifice, only 7/9 mice in the treated group reached 100% mammary gland tumor incidence as compared to 11/11 in the control group, translating into an overall extended survival upon treatment (Fig. 5B) (see also Additional file: Tab. S2). Moreover, Bio-nFeR effectively inhibited the growth of primary mammary tumors (Fig. 5C and E). The average tumor volume at sacrifice was 510.3 ± 88.6, versus 161.8 ± 59.3.3 (Fig. 5C), and the average tumor weight was 0.271 ± 0.054 vs. 0.112 ± 0.041 (Fig. 5E) (see also Additional file: Tab. S2). Altogether, Bio-nFeR treatment resulted in a reduction of overall tumor burden/mice at sacrifice (Fig. 5D and F). Mammary tumors explanted post-sacrifice did not display evident differences upon H&E histological analysis (Additional file: Fig. S3A). However, a lower frequency of Ki67-positive, proliferating cells, was observed in treated *versus* control tumors by immunofluorescence (Fig. 5G). Finally, analysis of DNA fragmentation by Terminal deoxynucleotidyl Transferase (TdT) dUTP Nick-End Labeling (TUNEL) assay did not reveal statistically significant differences between treated and untreated tumors, although some TUNEL-positive spots were present in Bio-nFeR-treated samples (Additional file: Fig. S3B). Taken together, these observations indicate that Bio-nFeR inhibits BC initiation and progression by inhibiting tumor cell proliferation and motility.

### Bio-nFeR reduces stem/progenitor cell content in breast tumors

**Fig. 6:**
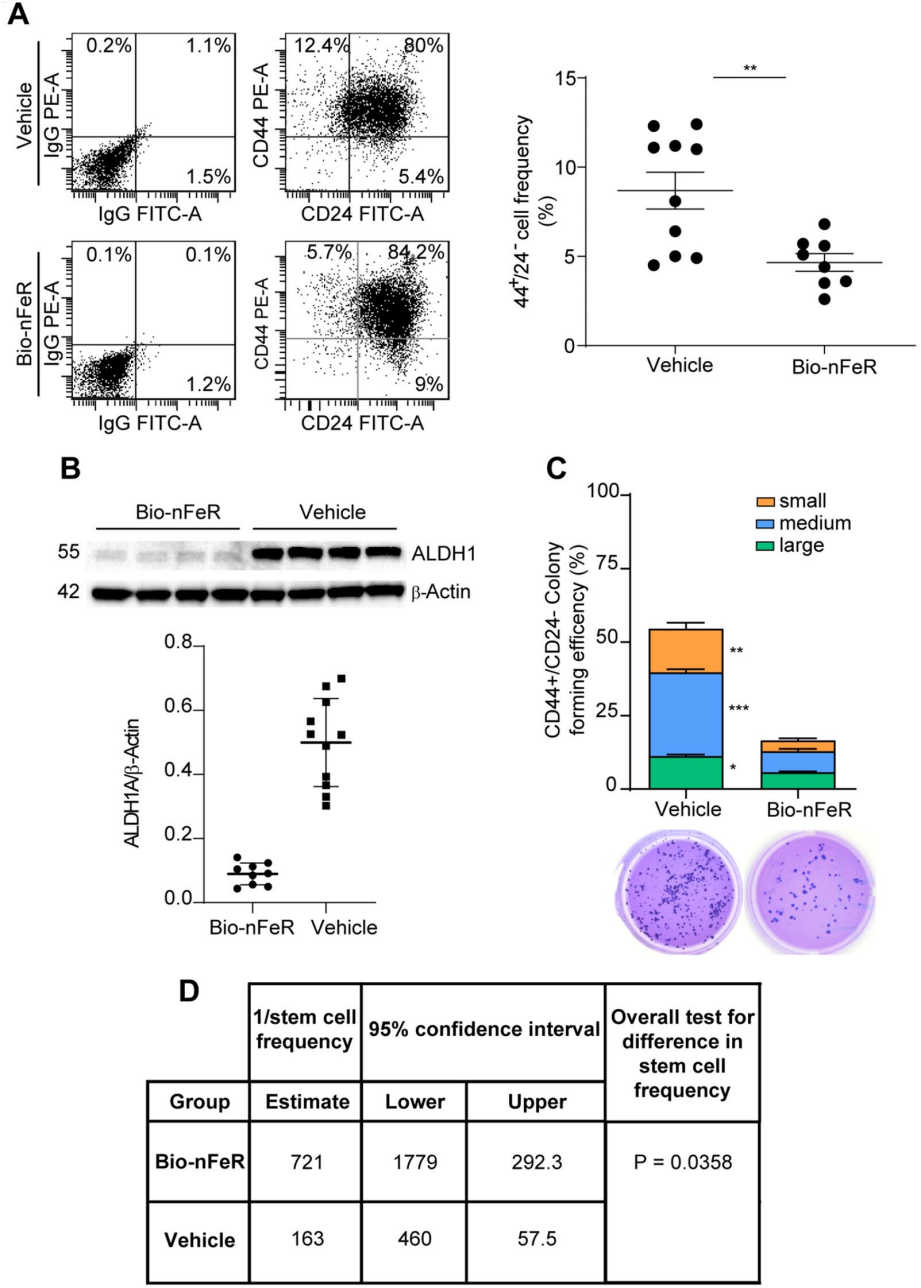
Bio-nFeR targets BC stem cells within neuT mice tumors. **(A)** Flow cytometry analysis showing the frequency of Lin^neg^/CD44^+^/CD24^−^ cells in Bio-nFeR-treated and vehicle-treated mice. Left: representative plots; Right CD44^+^/CD24^−^ quantification. **(B)** Upper panel: immunoblot analysis of ALDH1 on whole lysates of tumors harvested from Bio-nFeR treated and vehicle mice (see also Additional file: Fig S4A). β-actin was used as a loading control. Lower panel: ALDH1 quantification. **(C)** Self-renewal capacity of cells isolated from tumors as in A, evaluated as colony formation in semisolid culture and expressed as normalized colony size/percentage over plated cells. Values represent the mean ± SD of three technical replicates. *P < 0.05, **P < 0.01 and ***P < 0.001 by unpaired Student’s t test. **(D)** Limiting dilution assay by second transplantation into NSG mice, demonstrating a lower content of stem cells into treated tumors. Tumor-initiating cell assay performed on cells dissociated from Bio-nFeR-treated and vehicle-treated mice tumors was evaluated through second transplantation into NSG mice and quantified with the Extreme Limiting Dilution Analysis (ELDA) [29], software. Six mice were used for each dilution point. *P < 0.05

According to previous studies, FeR is able to affect the CSCs compartment both in vitro and in cancer xenografts [8, 9, 34]. To evaluate CSCs content following Bio-nFeR treatment, mammary tumors harvested at sacrifice were dissociated into single cells and analyzed by different methods (Fig. 6). Flow cytometry assessment of CD44 and CD24 expression ex vivo showed that Bio-nFeR induced a decrease in the CD44^+^/CD24^−^ CSCs population (Fig. 6A ). Moreover, immunoblot analysis of whole tumor lysates showed a lower expression of the stem cell marker ALDH1 in Bio-nFeR-treated samples (Fig. 6B and Additional file: Fig. S4A). Since the expression of CSCs markers may not be sufficient to evaluate the presence of tumorigenic cells, we investigated CSCs functional properties such as colony formation and tumor initiation. To this end, tumor cells isolated ex vivo as described above were plated in soft agar and the frequency of clonogenic units was evaluated after three weeks. Bio-nFeR treatment of mice strongly decreased the overall frequency of clonogenic cells in ex vivo samples with maximal efficacy on medium-sized colonies (Fig. 6C). These observations prompted us to investigate whether Bio-nFeR treatment was able to affect the frequency of tumor-initiating cells in vivo. To this end, we used freshly dissociated tumor cells from treated and control mice to perform a limiting dilution assay, consisting of subcutaneous secondary transplantation of defined numbers of BC cells into immunocompromised (NSG) recipient mice. Stem cell frequency was then calculated by the Extreme Limiting Dilution Assay (ELDA) software [29], and resulted significantly lower in samples derived from neuT mice treated with Bio-nFeR, indicating that Bio-nFeR reduces the frequency of tumorigenic cells within mammary tumors (Fig. 6D and Additional file: Tab. S3). The lower stem cell content detected in Bio-nFeR-treated tumors is fully consistent with the delayed/reduced tumor incidence observed in Bio-nFeR-treated mice (Fig. 5A-B), providing further support for possible use of Bio-nFeR as a chemopreventive drug.

### Bio-nFeR inhibits tumor growth by acting on cellular proliferative, metabolic and biosynthetic pathways

**Fig. 7:**
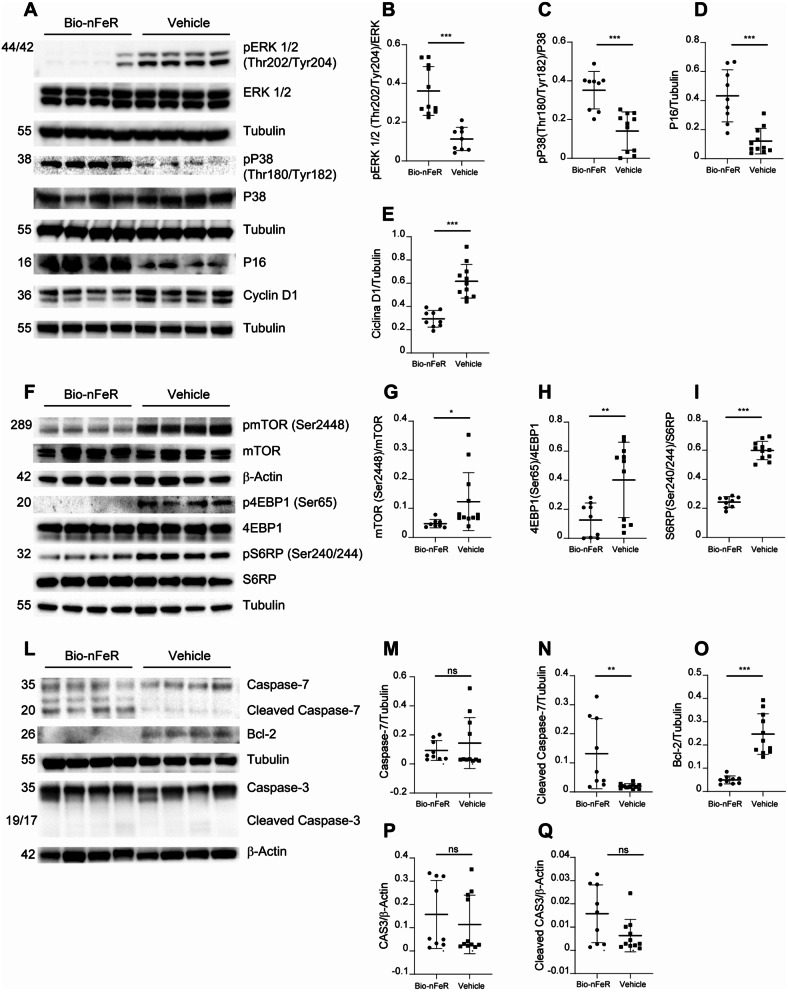
Protein expression analysis of Bio-nFeR versus vehicle mice tumors ex vivo. **A-E)** Left: Immunoblot analysis of cell cycle regulators ERK1/2, phospho-pERK1/2, p38, phospho-p38, p16 and cyclin D1 on Bio-nFeR versus vehicle mice tumors ex vivo (see also Additional file: Fig. S4B). Tubulin was used as a loading control. Right: quantification of the immunoblot shown on the left. **F-I)** Left: Immunoblot analysis of metabolic mTOR pathway components mTOR, phospho-mTOR, 4EBP1, phospho-4EBP1, S6RP and phospho-S6RP on Bio-nFeR versus vehicle mice tumors ex vivo (see also Additional file: Fig. S4C). Tubulin and β-actin were used as a loading control. Right: quantification of immunoblot shown on the left. **L-Q)**; Left: Immunoblot analysis of cell death-related proteins Caspase 7, Caspase 3 and Bcl-2 on Bio-nFeR versus vehicle mice tumors ex vivo (see also Additional file: Fig. S4D). Tubulin and β-actin were used as a loading control. Right: quantification of the immunoblot shown on the left

We and others previously showed that FeR and its derivatives affect multiple molecular pathways involved in cell proliferation, viability, metabolism, and biosynthesis [8, 10, 18]. To assess whether similar mechanisms of action occurred also in BC upon Bio-nFeR treatment, we performed immunoblot analysis of mammary tumors ex vivo for key factors involved in cellular proliferation and biosynthesis such as pERK, p38, p16, Cyclin D1, mTOR, 4EBP1, and S6RP (Fig. 7 and Additional file: Fig. S4B-D). Tumors treated with Bio-nFeR showed a strongly increased expression of phosphorylated p38 as compared to untreated tumors, and a decreased amount of phosphorylated pERK, where the low pERK/p38 ratio is indicative of cancer dormancy [35]. Bio-nFeR-treated tumors showed a decreased expression of Cyclin D1 and increased levels of p16, as compared to controls, further supporting a pro-dormancy action of Bio-nFeR treatment (Fig. 7A-E and Additional file: Fig. S4 B). Bio-nFeR-treated tumors showed a strongly reduced activation of mTOR and its downstream effectors 4EBP1 and S6RP (Fig. 7F-I and Additional file: Fig. S4C), in agreement with our previous data [8], and consistently with the inhibitory effect of the drug on cell metabolism [36]. Then, we investigated the levels and activation of proteins involved in apoptosis such as caspase-3, caspase-7, and Bcl-2 (Fig. 7L-Q and Additional file: Fig. S4D). Surprisingly, we detected cleaved caspase-7 but not cleaved caspase-3 in Bio-nFeR-treated tumors. This picture is consistent with the absence of apoptosis but the occurrence of cell cycle arrest, as explained in the Discussion. Finally, we found strongly reduced Bcl-2 levels in tumors treated with Bio-nFeR. Bcl-2 downregulation can indicate an increased sensitivity to death-inducing stimuli but is also linked to decreased metabolic resilience and energy production independently of cell death [37]. Altogether, these observations indicate that Bio-nFeR treatment counteracts tumor growth by inducing cellular dormancy together with a depression of metabolic and biosynthetic pathways.

### Bio-nFeR induces metastatic dormancy in tumor-bearing neuT mice

**Fig. 8:**
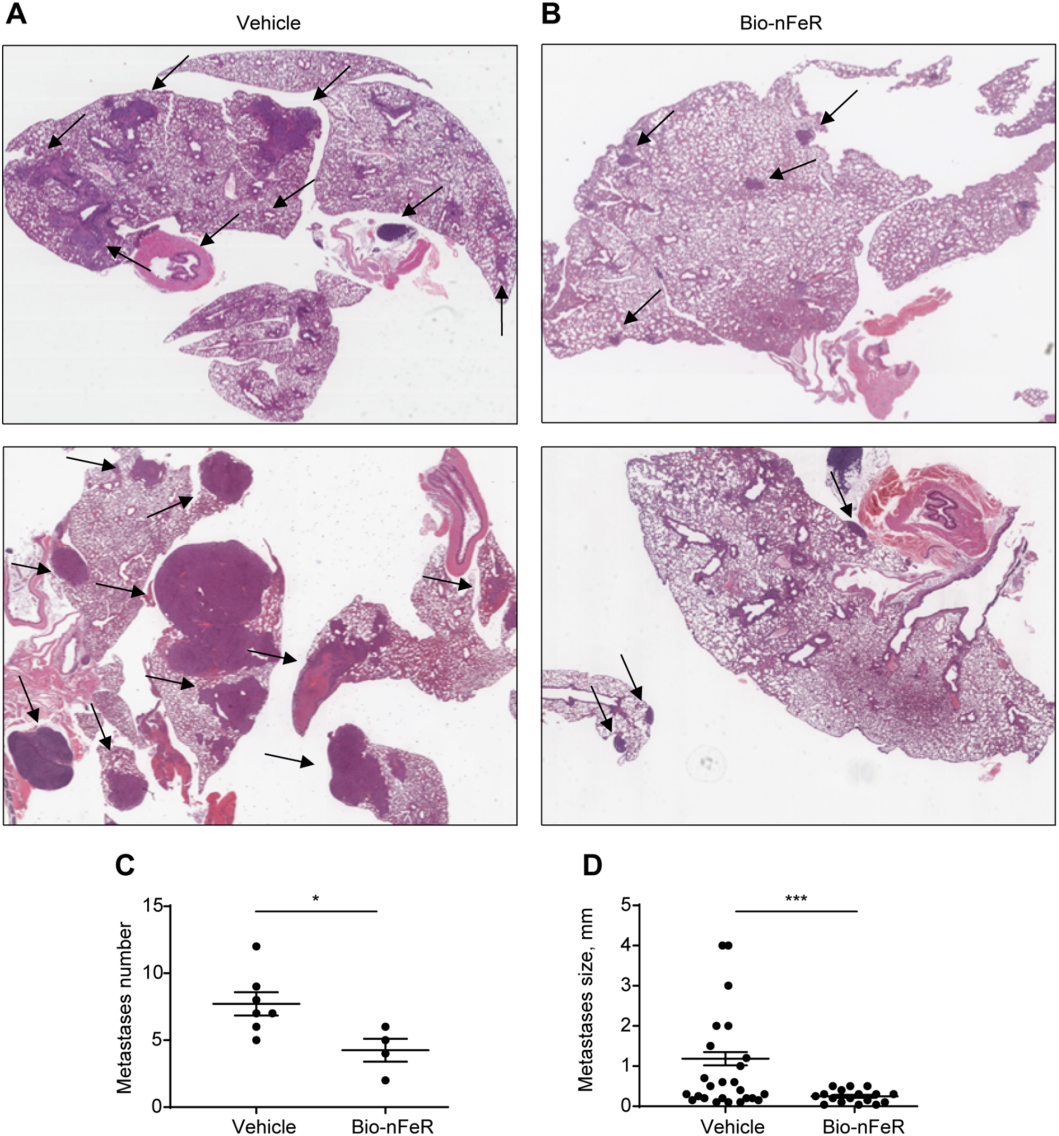
Bio-nFeR treatment reduces the initiation and growth of lung metastases in neuT mice. **A-B)** Whole sections of mice lungs showing BC lung metastases (arrows) of different sizes and numbers occurring in the pulmonary parenchyma of .Bio-nFeR-treated versus control mice (H&E sections, 1x, digital picture Aperio ImageScope). **C)** Average metastases number within metastasis-positive lungs in Bio-nFeR treated versus vehicle mice (scatter plot). **D)** Average metastases size in Bio-nFeR treated versus vehicle mice (scatter plot). Data represent Mean ± SEM, *P < 0.05 and ***P < 0.001 by unpaired Student’s t test with Welch’s correction

**Fig. 9:**
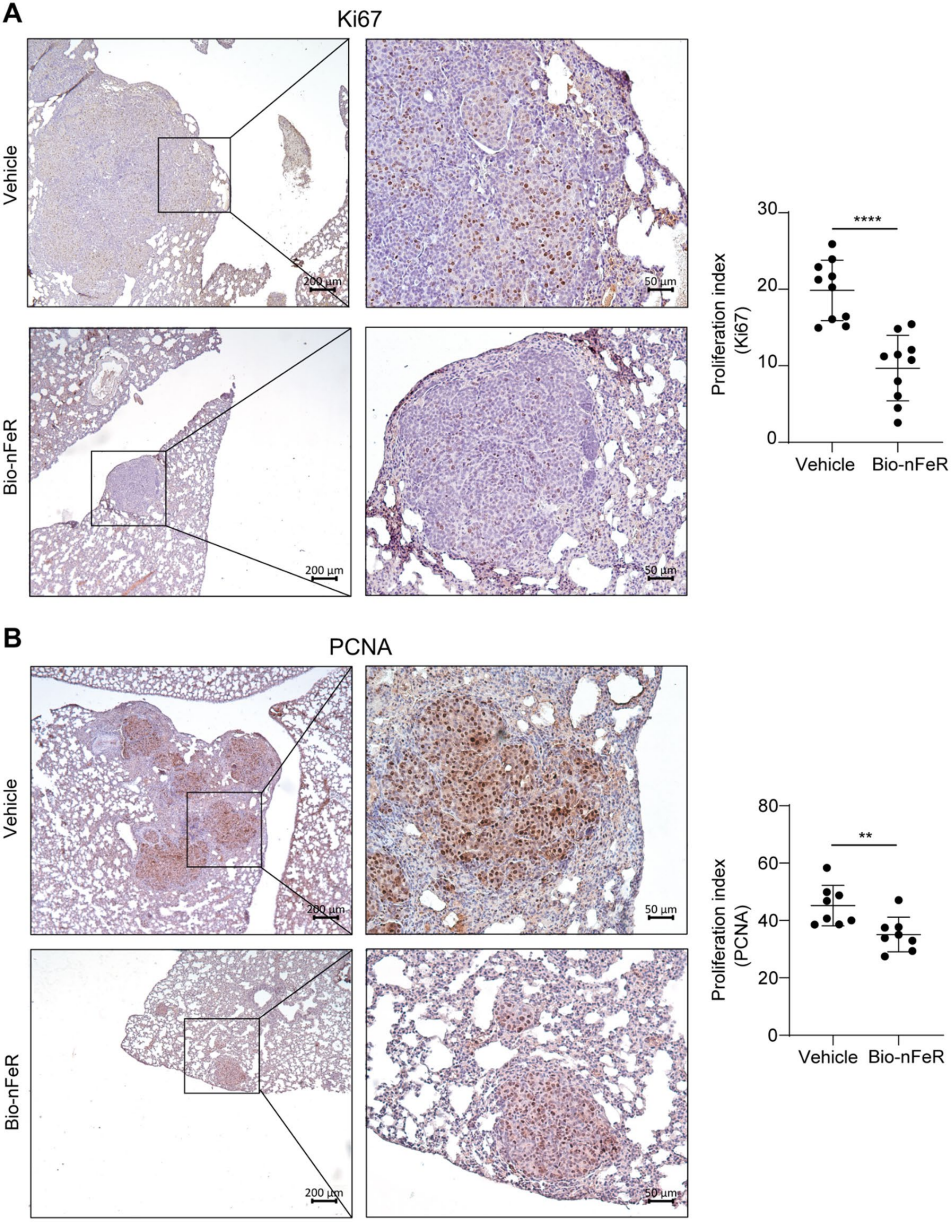
Immunohistochemistry analysis of Ki67 and PCNA on lung metastasis from Bio-nFeR versus control mice. **(A)** Left: Representative IHC images of Ki67-positive nuclei on lung metastasis from Bio-nFeR versus vehicle mice; Right: Proliferation index by Ki67 staining in treated and control metastases; **(B)** Left: Representative IHC images of PCNA-positive nuclei in lung metastases from Bio-nFeR versus vehicle mice; Right: Proliferation index by PCNA nuclear staining in treated and control metastases. Quantifications were obtained by analyzing images at 20x magnification. Proliferation index was calculated by automated pixel counting, as the ratio between Ki67-or PCNA-positive/total nuclei (see Methods and Additional file: Fig. S5). Magnifications 5x and 20x, scale bares 200 and 50 μm, respectively

In 129 Sv-neuT mice, mammary adenocarcinomas spontaneously generate lung metastases beginning 24 weeks PB (unpublished observations). To evaluate the effect of Bio-nFeR on BC metastasis initiation and progression, we analyzed lungs from treated and untreated mice harvested at sacrifice. Lung metastases of BC were present in 4/9 Bio-nFeR-treated and 7/11 control mice. Representative H&E images of metastatic lungs from untreated versus Bio-nFeR-treated mice are shown in Fig. 8A-B. Sections of metastases-positive lungs were analyzed for tumor frequency and size as described in Materials and Methods (Fig. 8C-D and Additional file: Tab. S2). The average frequency of metastases was nearly halved in Bio-nFeR-treated versus control animals (4.25 ± 0.85 vs. 7.7 ± 0.86, Fig. 8C, see also Additional file: Tab. S2). Importantly, the average size of metastatic foci was significantly smaller in Bio-nFeR-treated versus control mice (0.25 ± 0.04 vs. 1.2 ± 0.16 mm, Fig. 8D, see also Additional file: Tab. S2), suggesting that Bio-nFeR exerts its strongest effect in counteracting the growth of metastatic breast tumors. To investigate the proliferative status of metastatic cells in the lungs of Bio-nFeR-treated and control mice, we performed immunohistochemical staining of metastatic lungs for Ki67 (Fig. 9A and Additional File: Fig. S5) and Proliferating Cell Nuclear Antigen (PCNA) (Fig. 9B and Additional file: Fig. S5). For each of the two proliferation markers, equivalent areas of metastatic tissue were used to quantify proliferation marker-stained nuclei over total nuclei (see Methods and Additional File: Fig. S5). In line with the smaller size of lung metastases in Bio-nFeR-treated mice, both Ki67 and PCNA expression was strongly reduced in metastatic BC cells, indicating that Bio-nFeR induces metastatic dormancy (Fig. 9B-D). Taken together, the results of functional CSCs assays and metastasis quantification show that Bio-nFeR counteracts BC metastatic progression by inhibiting metastasis-founder cells (MFCs) and by promoting the dormancy of metastatic tumors.

## Discussion

Retinoids are synthetic and natural vitamin A derivatives that have been widely investigated for the treatment of solid and hematological tumors [38]. Retinoic acid (RA) therapy has been successfully used in acute promyelocytic leukemia and childhood neuroblastoma [39]. By contrast, RA treatment of solid tumors generally led to controversial results, mainly because of problems related to drug solubilization, photosensitivity and unwanted side effects [39, 40]. Among retinoids, FeR has been regarded for long as a promising antitumor agent due to its ability to induce cancer cell differentiation, death, and growth arrest [10]. However, the scarce solubility and bioavailability of FeR prevented to achieve plasma concentrations within the therapeutic window observed in vitro. Recent attempts to improve FeR bioavailability include intravenous administration of the drug in lipid emulsions, achieving therapeutic plasma concentrations [41, 42] and promising clinical responses in lymphoid malignancies [42]. However, such FeR formulation showed minimal evidence of effectiveness on solid tumors in Phase I studies both as a single agent and in combination [41, 43]. Bio-nFeR is an improved FeR formulation based on drug encapsulation in an ion-pair stabilized lipid matrix [9]. Our previous studies showed that Bio-nFeR is characterized by high aqueous solubility and increased oral absorption, presenting as an effective candidate for future clinical studies. Bio-nFeR showed antitumor activity against melanoma, lung, and colorectal cancer xenografts with a specific action against CSCs [9]. The effectiveness of Bio-nFeR in preclinical models of solid tumors prompted us to investigate its potential activity in BC. To investigate Bio-nFeR effects on BC development, progression, and metastasization, we chose the neuT mouse model as it recapitulates all the main steps of BC evolution avoiding the drawbacks of xenograft models. In fact, Bio-nFeR treatment resulted in a reduced incidence and size of both primary mammary tumors and pulmonary metastases in neuT mice. These observations are consistent with a capability of Bio-nFeR to target CSCs [8, 9, 34]. Accordingly, tumors of Bio-nFeR-treated mice showed a contraction of the CD44^+^/CD24^−^ population, a reduced expression of stem cell-associated ALDH1, lower content of colony-forming units, and decreased frequency of tumor-initiating cells upon second transplantation in immunocompromised mice. Besides its CSCs-targeting action, we found that Bio-nFeR could exert inhibitory effects on the main pathways responsible for cell proliferation and biosynthesis. Specifically, we observed a low pERK/p38 ratio, low expression of cyclin D1 and increased levels of p16 in Bio-nFeR-treated tumors, which cumulatively indicate a dormancy state. Moreover, we observed a generalized depression of the mTOR pathway, which is generally regarded as a hallmark of dormancy in cancer cells [44]. Importantly, however, while cell cycle slowdown and mTOR repression are usually associated with increased stemness and regenerative potential [5, 44], Bio-nFeR seems to establish a dormancy state characterized by cell quiescence and decreased stem cell content. This property of Bio-nFeR is particularly important when considering long-term potential outcomes, as treatments that potentiate the CSC compartment may promote tumor relapse both in solid and hematological cancers [45–48]. Bio-nFeR mechanism of action in BC cells did not involve a significant activation of apoptosis pathways, as indicated by the absence of cleaved caspase-3 in treated tumors. Intriguingly, however, we found that Bio-nFeR induced caspase-7 cleavage/activation, which could be involved in mediating Bio-nFeR effects through two different mechanisms. First, caspase-7 has been reported as the only caspase involved in cell cycle regulation [49]. In BC cells, caspase-7 acts through p21^cip1/waf1^ to regulate cell cycle progression or arrest [50]. Additionally, activated caspase-7 has been reported to regulate cell cycle regulatory factors such as claspin and YY1 [51, 52]. Secondly, caspase-7 has been recently shown to activate the acidic sphingomyelinase resulting in ceramide production and preservation of cell membrane integrity to delay immune-mediated cell death [53]. As FeR activates sphingolipid metabolism and ceramide generation [54], caspase-7 may be involved in mediating the multiple effects of ceramide on Bio-nFeR-treated tumors. The anticancer effects of Bio-nFeR were evident particularly in the metastatic context. In fact, the reduced frequency and the decreased proliferation of pulmonary metastases indicate that Bio-nFeR treatment effectively inhibits metastasis-forming cells. Metastasis inhibition may be due to a reduced frequency of metastasis-initiating cells in primary tumors, downregulation of BC invasive capacity, and/or a block of metastatic cell expansion at a very early stage. It is likely that these effects occur simultaneously, resulting in a consistent and homogeneous reduction of metastasis size as compared to untreated animals. These findings are partially in line with the results of a fifteen-year phase III clinical trial of FeR for relapse prevention in BC patients completed in 2006. This study showed a significant reduction of tumor recurrence in premenopausal women upon FeR treatment [17], indicating an effect on relapse-inducing BC cells. However, clinical trial results showed that FeR was effective only in preventing local relapse while it did not show any efficacy against distant metastases [17]. This limitation may be due to the scarce bioavailability of FeR used in the clinical study, suggesting that an improved bioavailability and increased plasma concentrations may be crucial for achieving antimetastatic effects in both preclinical and clinical settings. The virtual absence of Bio-nFeR toxic effects on healthy tissues and organs is particularly interesting in light of the potential use of this drug as a chemopreventive agent in BC patients at high risk of developing metastatic disease. Such a clinical setting would require a prolonged administration schedule that must be compatible with the occurrence of unwanted side effects. Our studies showed that Bio-nFeR and its previous formulation nFeR did not cause hepatic toxicity, hematological toxicity, or weight loss in treated mice [8, 9]. Moreover, prolonged treatment of mice with either nFeR (8–9 weeks) or Bio-nFeR (12–16 weeks) was well tolerated and effectively inhibited tumor progression [8, 9], indicating Bio-nFeR as a potential candidate for BC chemoprevention.

## Conclusions

Taken together, the ability of Bio-nFeR to inhibit BC cell proliferation, to reduce CSCs content and to counteract both primary and metastatic BC progression in the absence of toxic side effects indicate this agent as a potential candidate for BC treatment and metastasis prevention.

## Electronic supplementary material

Below is the link to the electronic supplementary material.

Supplementary Material 1

## Data Availability

The datasets generated and/or analysed during the current study are available from the corresponding author on reasonable request.

## Abbreviations

ALDH1: Aldehyde dehydrogenase 1
Bio-nFeR: Bionanofenretinide
BWL: Body weight loss
BC: Breast cancer
CRC: Colorectal cancer
CSCs: Cancer stem cells
FACS: Fluorescence-activated cell sorting
FeR: Fenretinide
Ki67: Antigen Kiel 67
MFCs: Metastasis founder cells
neuT: HER2/neu transgenic mice
nFeR: Nanofenretinide
NSG: NOD/SCID non-obese diabetic/severe combined immunodeficiency gamma chain deficient
PB: Post birth
PCNA: Proliferating Cell Nuclear Antigen
RA: Retinoic acid
ROI: Region of Interest
TUNEL: Terminal deoxynucleotidyl transferase dUTP nick end labeling
