# Supplementary material for: A nanoencapsulated oral formulation of fenretinide promotes local and metastatic breast cancer dormancy in HER2/neu transgenic mice
Source: J Exp Clin Cancer Res. 2024 Nov 5;43:296. doi: 10.1186/s13046-024-03213-6 (PMC11536529; doi:10.1186/s13046-024-03213-6)
Supplement: Supplementary file 1 — Supplementary Material 1 [file 13046_2024_3213_MOESM1_ESM.pdf]

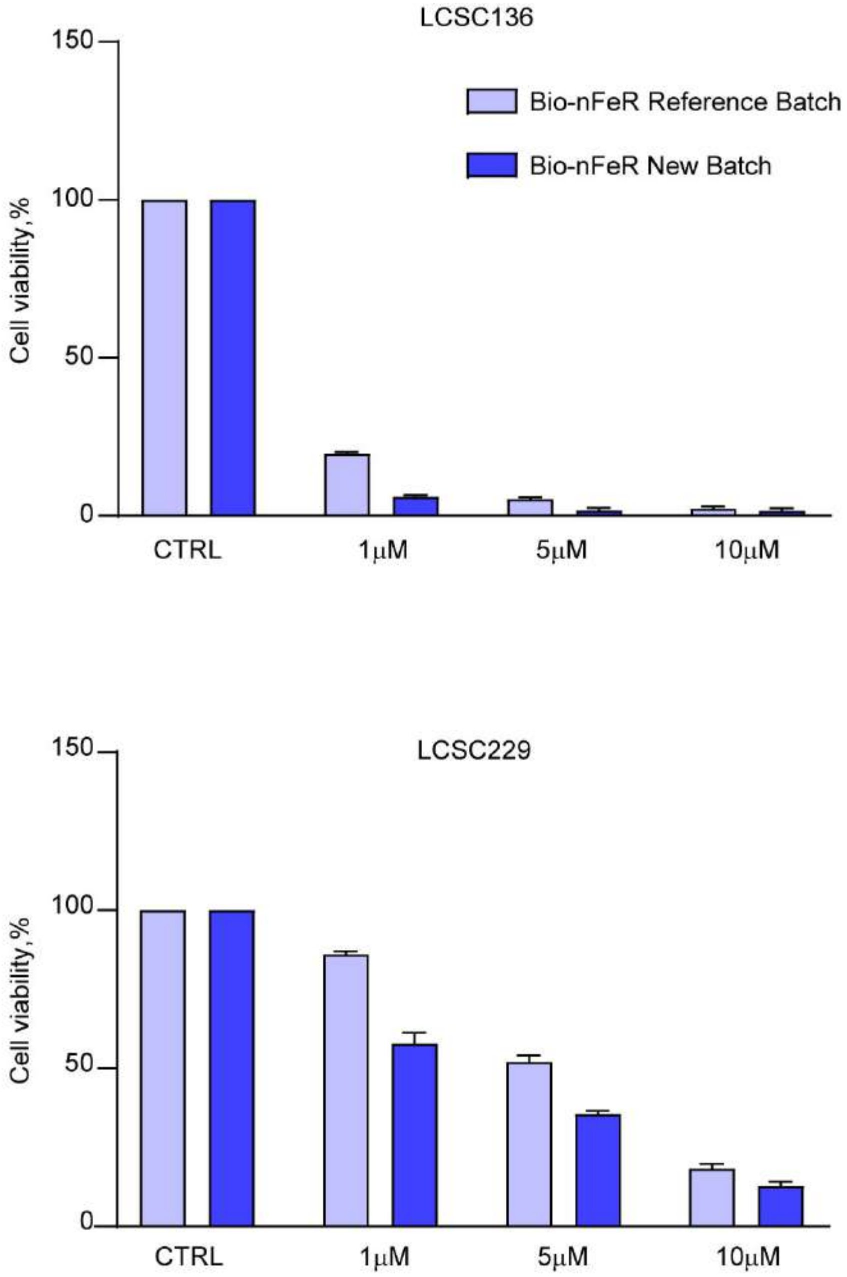

Supplementary Figure S1. Cell viability inhibition test of Bio-nFeR batch used in this study versus data from the Reference Lot in (9). Lung cancer stem cell lines LCSC136 and LCSC229 were treated at the indicated concentrations, for 72 hours. Values represent the mean ± SD of three independent experiments.

A

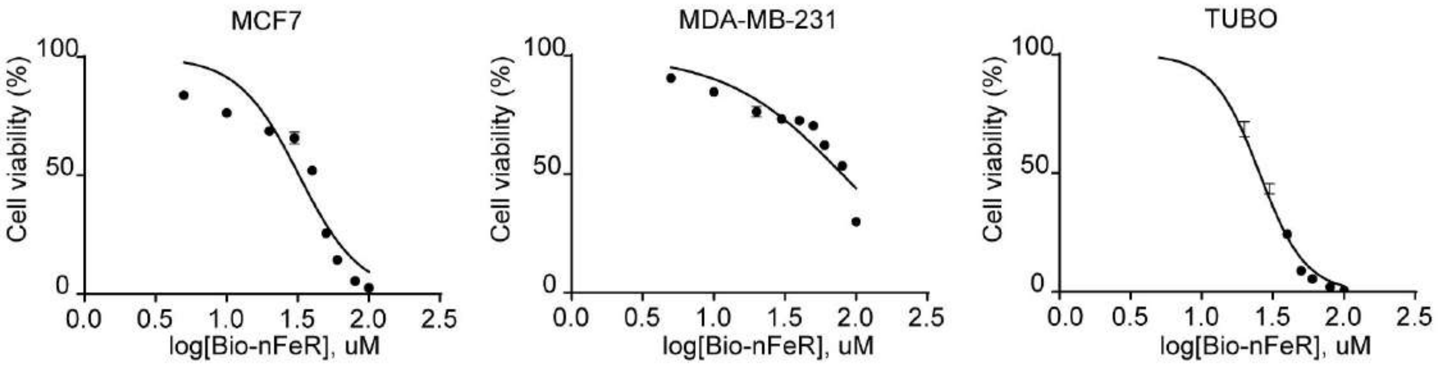

Bottom	= 0.000
Top	= 100.0
LogEC50	1.510
HillSlope	-2.019
EC50	32.37

Bottom	= 0.000
Top	= 100.0
LogEC50	1.903
HillSlope	-1.065
EC50	79.93

Bottom	= 0.000
Top	= 100.0
LogEC50	1.407
HillSlope	-2.682
EC50	25.53

Supplementary Figure S2. Determination of Bio-NFeR IC50 on MCF7, MDA-MDB-231 and TUBO cells, treated as in Fig. 1A, by GraphPad prism software.

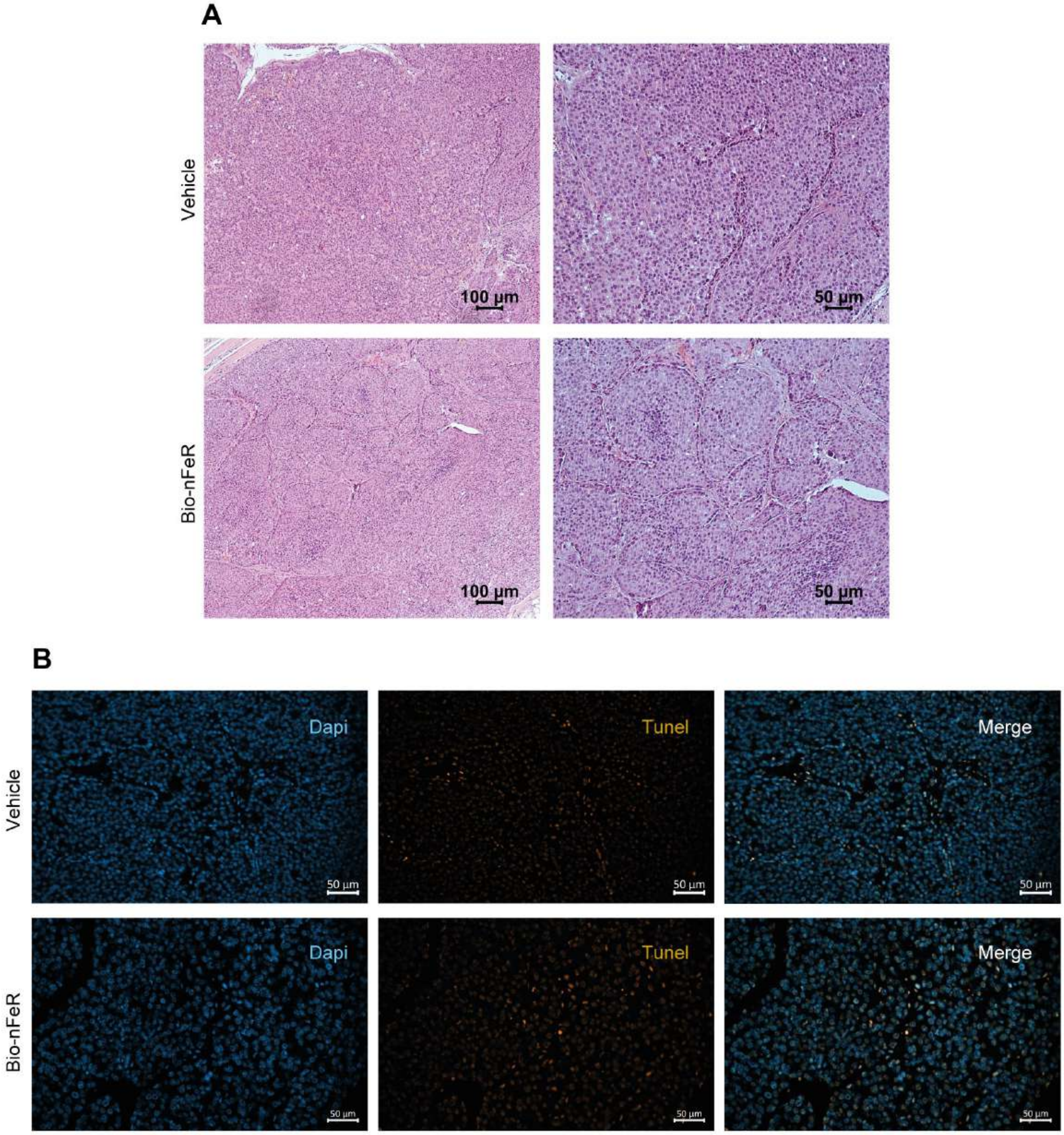

Supplementary Figure S3. A) Representative H&E images of primary mammary tumors harvested *ex vivo* from Bio-nFeR and control mice; B) Representative image of TUNEL assay on treated and control tumors.

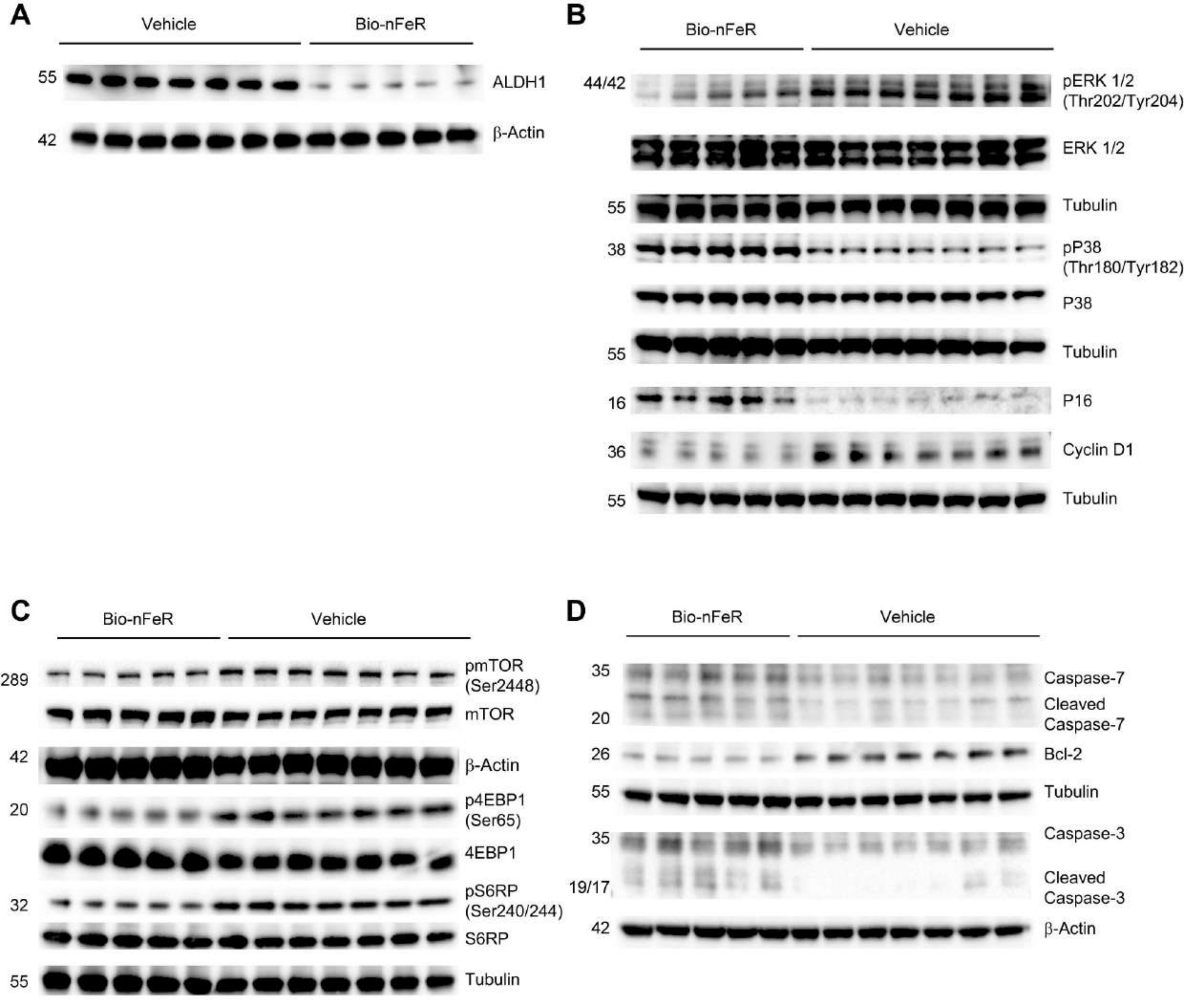

Supplementary Figure S4. Western blot analysis of Bio-nFeR versus vehicle mice tumors ex vivo: A: blots not shown in Fig. 6A; B-D: blots not shown in Fig. 7.

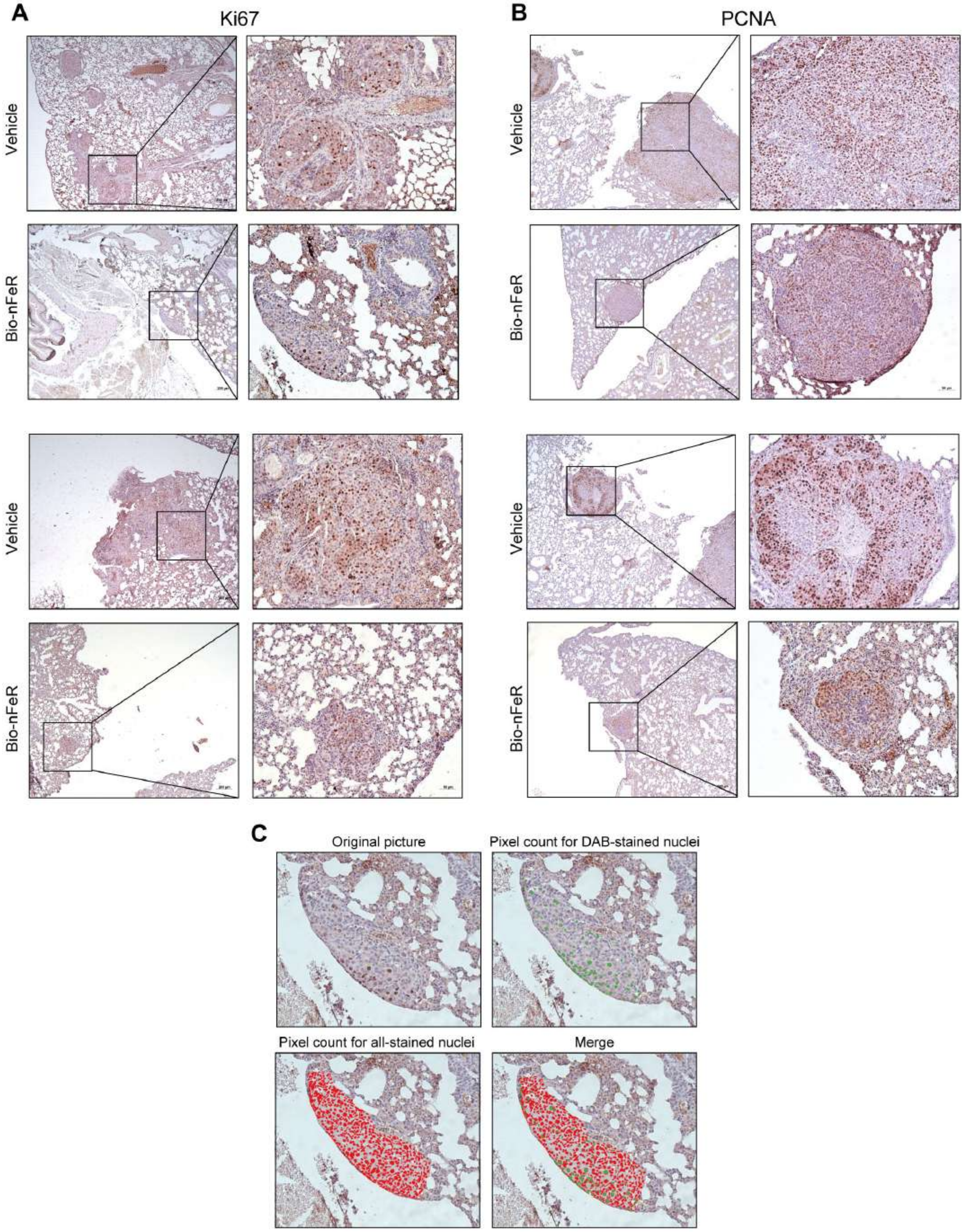

Supplementary Figure S5. Proliferation index analysis of Ki67 A) and PCNA B) on IHC images of lung metastases in Bio-nFeR versus control mice (see Fig. 9). Representative images are shown: Magnification 5X and 20X. C) Representative image of computer-assisted analysis of stained slides with ZEN 2.6 (blue edition) software. Thresholds were set within ROIs before performing quantification. Upper left: Original image; Upper right: area of marker-stained nuclei (DAB, pseudocolored in green); Lower left: Area of total nuclear staining (hematoxylin, pseudocolored in red); lower right: Merge. Proliferation index (Fig. 9 B and D) was calculated by the software as the percentage of the green over the red area.

Supplementary Table S1. Evaluation liver A) and blood toxicity B), and plasma concentration C), and liver in mice after 2 weeks oral daily treatment at dose of 100 mg/kg of Bio-nFeR formulation or vehicle. A) Hepatic enzyme determination AST: Aspartate transaminase; ALT: Alanine transaminase; Bil: Bilirubin total; Data represent mean ± SEM; B) Hematic values: Bilirubin and blood cell counts; C) Plasma concentration of fenretinide and fenretinide metabolites.

A

	**AST (U/L)**	**ALT (U/L)**	**Bil (mg/dl)**
**Bio-nFeR**	108 ± 9	34,5 ± 4,5	0,37 ± 0,04
**Vehicle**	66,5 ± 0,5	21 ± 3	0,475 ±0,05

B

**Parameters**		**Bio-nFeR**		**Vehicle**	
		mean	SEM	mean	SEM
RBC	$x10^6$	9,25	2,05	9,095	1,905
HB	g/dl	15,1	1,8	16,65	1,35
HCT	%	37,15	1,85	38,05	1,95
MCV	Fl	47,5	1,5	53,3	2,3
MCH	Pg	16,65	1,75	18,75	2,45
MCHC	g/dl	32,5	0,65	32	3,1
CHCM	g/dl	31,65	0,64	32	0
RDW	%	13,3	1	14,95	0,75
HDW	g/dl	2,3	0,2	2,05	0,15
WBC	$x10^3$/µl	5,7	0,4	4,2	1,4
Neutrophils	$x10^3$/µl	0,815	0,37	0,83	0,25
Lymphocytes	$x10^3$/µl	4,505	0,06	3,045	1,175
Monocytes	$x10^3$/µl	0,09	0,09	0,085	0,025
Eosinophils	$x10^3$/µl	0,17	0,01	0,14	0,025
Basophils	$x10^3$/µl	0,005	0,005	0,005	0,005
PLT	$x10^3$/µl	1027	18	1014	16
MPV	fl	12,6	3	11,7	4

C

**Treatment**	**Mean plasma concentration ± SEM (ng/mL)**			
	**4HPR**	**4HPR-DH**	**4HPR-DH-OH**	**4HPR-CH3**
**Bio-nFeR**	1010,8±111,5	410,9±99,6	255.3±47,5	156.1±39,5
**Vehicle**	ND	ND	ND	ND

Supplementary Table S2. Effects of Bio-nFeR on tumor and lung metastasis development in NeuT mice. Data from two independent experiments are reported. Data from experiment 2 are described in detail in Fig. 5 (panels A-F), Fig. 6 (panel D), and Fig. 8.

**EXPERIMENT 1**			
	**Vehicle**	**Bio-nFeR**	
Mice	6	6	
Total tumors at sacrifice (28w PB)	60	54	
Average tumor vol at sacrifice (28w PB), mm3	378±109	128±36	P=0.008
#metastatic mice	3/6	1/6	
Average metastasis #/metastatic mouse	9	6	ns
Metastasis average size	0,9 ± 0,1	0,2 ± 0,03	P=0.001
**EXPERIMENT 2**			
	**Vehicle**	**Bio-nFeR**	
Mice	11	9	
Average tumor/mouse at sacrifice (32w PB)	110	83	
Average tumor vol at sacrifice (32w PB), mm3	498 ± 142	98 ±24	0.003
#metastatic mice	7/11	4/9	
Average metastasis #/metastatic mouse	7.7±0.86	4.25±0.85	0.014
Metastasis average size	1.2±0,16 mm	0,25±0,04	0.0006

Supplementary Table S3: Limiting dilution by tumor cell transplantation into secondary NSG mice.

Group	Treatment	Cell dose	Mice tested	Mice positive
1	**Vehicle**	1000	6	6
2	**Vehicle**	100	6	3
3	**Vehicle**	10	6	0
4	**Vehicle**	1	6	0
5	**Bio-nFeR**	1000	6	5
6	**Bio-nFeR**	100	6	0
7	**Bio-nFeR**	10	6	0
8	**Bio-nFeR**	1	6	0
